# Quantitative Trait Loci Affecting Liver Fat Content in Mice

**DOI:** 10.1534/g3.112.003343

**Published:** 2012-09-01

**Authors:** Olga Minkina, James M. Cheverud, Gloria Fawcett, Clay F. Semenkovich, Jane P. Kenney-Hunt

**Affiliations:** * Department of Anatomy and Neurobiology, and; † Department of Medicine, Washington University School of Medicine, St. Louis, Missouri 63110

**Keywords:** nonalcoholic fatty liver disease, NAFLD, QTL, mouse, LG/J, SM/J

## Abstract

Nonalcoholic fatty liver disease, a condition in which excess fat accumulates in the liver, is strongly associated with the metabolic syndrome, including obesity and other related conditions. This disease has the potential to progress from steatosis to steatohepatitis, fibrosis, and cirrhosis. The recent increase in the prevalence of the metabolic syndrome is largely driven by changes in diet and activity levels. Individual variation in the response to this obesogenic environment, however, is attributable in part to genetic variation between individuals, but very few mammalian genetic loci have been identified with effects on fat accumulation in the liver. To study the genetic basis for variation in liver fat content in response to dietary fat, liver fat proportion was determined using quantitative magnetic resonance imaging in 478 mice from 16 LG/J X SM/J recombinant inbred strains fed either a high-fat (42% kcal from fat) or low-fat (15% kcal from fat) diet. An analysis of variance confirmed that there is a genetic basis for variation in liver fat content within the population with significant effects of sex and diet. Three quantitative trail loci that contribute to liver fat content also were mapped.

Components of the metabolic syndrome, including obesity, type 2 diabetes, insulin resistance, and hyperdyslipidemia, are very frequently accompanied by nonalcoholic fatty liver disease (NAFLD) (Del Gaudio *et al.* 2002; Marchesini *et al.* 2003; Adams *et al.* 2005; Fabbrini *et al.* 2009; Bugianesi *et al.* 2010). NAFLD describes a range of conditions varying in severity from steatosis to nonalcoholic steatohepatitis, to fibrosis and finally cirrhosis (James and Day 1998). Even in the less severe manifestations, such as steatosis, NAFLD has important consequences for health and metabolism. In several recent studies authors have shown that the accumulation of fat in the visceral cavity, and particularly in the liver, is primarily responsible for the negative metabolic effects of obesity (Stefan and Häring 2011).

Lipid accumulation in the liver is strongly correlated with insulin resistance, but it is currently unclear whether a causal relationship between insulin resistance and lipid accumulation in the liver exists (Fabbrini *et al.* 2009). A study of familial hypobetalipoproteinemia, in which a mutation impairs the ability to export intrahepatic triglycerides (IHTG), revealed that patients have similar levels of insulin sensitivity as patients with normal IHTG (Amaro *et al.* 2010). In addition, although a single-nucleotide polymorphism (SNP) in human *PNPLA3* is significantly correlated with increased hepatic fat, it is not correlated with insulin resistance (Romeo *et al.* 2008). Steatosis can also occur without insulin resistance in mice (Grefhorst *et al.* 2005; Minehira *et al.* 2008).

The relationship between lipid accumulation and body weight is similarly complex. Although NAFLD rates increase with increasing body mass index (Ruhl and Everhart 2003), not all obese subjects suffer from metabolic abnormalities. Conversely, some subjects with normal body mass index have these abnormalities (Wildman *et al.* 2008). Several studies argue that visceral adipose tissue is the most accurate predictor of the metabolic syndrome (Carey *et al.* 1996; Atzmon *et al.* 2002; Wajchenberg *et al.* 2002). Fabbrini *et al.* (2009), however, found that although visceral adipose tissue and IHTG measurements are correlated, IHTG content is the better predictor of metabolic abnormalities associated with obesity. Although even small amounts of weight loss in obese patients can reverse steatosis and reduce hepatic insulin resistance (Petersen *et al.* 2005; Adams and Angulo 2006; Klein *et al.* 2006; Larson-Meyer *et al.* 2008; Kirk *et al.* 2009), much remains unknown about the relationships between these metabolic dysfunctions or why some cases of steatosis progress to the more severe stages, whereas others do not.

The recent and rapid worldwide increase in metabolic syndrome and related conditions is thought to be attributable to changes in diet and activity level, resulting in an imbalance between energy intake and expenditure (Yanovski and Yanovski 1999; Heymsfield 2009). Individual response to this obesogenic environment is influenced by genetic variation between individuals (Musani *et al.* 2008). Metabolic syndrome and related conditions, including NAFLD, are complex traits, resulting from the expression of many alleles of small effect interacting with each other and with the dietary environment. Ethnicity and sex are known to play a role in the prevalence of NAFLD (Browning *et al.* 2004), suggesting complex interactions between genes, sex, and diet. To date, only a few genetic loci have been identified with effects on hepatic fat content in mammals (Rangnekar *et al.* 2006; Itoi-Babaya *et al.* 2007; Kumazawa *et al.* 2007; Romeo *et al.* 2008, Millward *et al.* 2009). Further understanding of the genetic factors that interact with the environment to contribute to the pathology of NAFLD will help to identify the physiologic pathways regulating lipid accumulation in the liver and aid in understanding the relationships between fatty liver disease and other obesity- and diabetes-related traits. Identification of quantitative trait loci (QTL) for hepatic fat accumulation in a nonmutant murine model of metabolic syndrome, such as the LG/J X SM/J intercross and derived strains (Ehrich *et al.* 2003; Cheverud *et al.* 2004a,b), can contribute substantially to our understanding of the genetics of NAFLD.

This experiment examined the genetic architecture of variation in lipid accumulation in the liver in response to dietary fat in 16 recombinant inbred mouse strains (RI strains) of the LG/J X SM/J intercross. Interactions between genetic background (strain), sex, and diet were identified. QTL analysis was performed to map QTL that contribute to liver fat content.

## Materials and Methods

The LG/J and SM/J inbred mouse strains (Jackson Laboratories, Bar Harbor, ME) were derived independently more than 60 years ago (Goodale 1938; Goodale 1941; MacArthur 1944). Selection for large and small body size at 60 days of age resulted in the creation of the LG/J and SM/J strains, respectively, with a mean body weight difference of 24 g at this age (Chai 1956; Chai 1957). Analysis of the genetic architecture of the body size of LG/J and SM/J strains revealed that the size differences between the two strains are attributable to the interaction of many genes of small effect (Chai 1956). Sixteen recombinant inbred strains (RI strains) obtained from the LG/J X SM/J F_2_ intercross were used in this analysis (Hrbek *et al.* 2006).

The mice were housed in a facility that maintained 12-hour light/dark cycles and a temperature of 21°. All procedures followed the guidelines for the care of laboratory animals at Washington University School of Medicine (Assurance #A-3381-01). Sires and dams from each of the 16 RI strains and LG/J and SM/J were fed a standard mouse chow (PicoLab Rodent Chow 20, #5053). Male and female offspring were weaned at 3 weeks of age, at which point they were separated into single-sex cages of no more than five animals. The mice were then fed *ad libitum* either a high-fat (42%, Harlan Teklad #TD88137) or low-fat (15%, custom, formerly Research Diets #D12284) diet. The diets are nearly isocaloric (Table 1). At 20 weeks of age, the mice were fasted for 4 hr in the morning, killed, and necropsied. At necropsy, the masses of the whole carcass, liver, fat pads (mesenteric, inguinal, reproductive, and kidney), heart, spleen, and kidney were collected. Blood plasma was obtained by cardiac puncture and analyzed for free fatty acids, cholesterol, triglycerides, leptin, and insulin content. Liver samples were stored at −80°.

**Table 1:** Components of high-fat (HF) and low-fat (LF) diets

	HF Diet	LF Diet
Energy from fat, %	42	15
Casein, g/kg	195	197
Sugars, g/kg	341	307
Corn starch, g/kg	150	313
Cellulose, g/kg	50	50
Corn oil, g/kg	−	58
Hydrogenated coconut oil, g/kg	−	7
Anhydrous milk fat, g/kg	210	−
Cholesterol, g/kg	1.5	−
Kilojoules per gram	18.95	16.99

Liver samples were thawed and placed into tubes that were inserted into an EchoMRI 3-in-1 instrument (Echo Medical Systems, Houston, TX) to measure liver fat content. The EchoMRI 3-in-1 is a nuclear magnetic resonance instrument that distinguishes between fat and lean content within tissues. Fat and lean content of each liver sample was measured in milligrams at the highest precision.

Quantitative magnetic resonance imaging (QMR) has a greater precision in determining fat and lean mass than dual-energy X-ray absorptiometry or chemical carcass analysis in studies of whole-body composition in mammals, and although slightly biased toward greater fat levels, is highly correlated to these other methods (Taicher *et al.* 2003; Tinsley *et al.* 2004; Napolitano *et al.* 2008; Johnson *et al.* 2009; Jones *et al.* 2009; Gallagher *et al.* 2010; Nixon *et al.* 2010). Because all of our data were collected with the same QMR protocol on the same machine, this bias does not have any effect on the variance among individuals or strains, which is the object of our study.

Additional data were collected to determine whether QMR data from these samples demonstrates high repeatability, high heritability, and high correlation to biochemical analysis. Holding tissues at −80°, thawing, and then measuring lipid levels with nuclear magnetic resonance may alter liver fat proportions relative to measures on fresh tissue. However, all specimens were treated the same, and it is the relative values, not the absolute ones, that are important for quantitative genetic analysis. Liver samples from a subset of high-fat fed males from this study (n = 35) were analyzed using chemical methods to determine hepatic triglyceride levels. QMR and chemical results were highly correlated both in phenotype (r = 0.69, *P* = 1 × 10^−6^) and in genotype (r = 0.73). Although QMR data showed a bias to greater values than results from the chemical analysis, the QMR data also had a greater heritability (H^2^ = 0.88), and data from genetically identical individuals were more similar to one another in QMR analysis than in chemical analysis. The higher heritability determined by QMR may be attributable in part to the very high precision of this method (Taicher *et al.* 2003; Tinsley *et al.* 2004). In an additional study of 20 archived liver samples from an outbred population, repeatability of fat mass and lean mass between QMR trials was excellent (r^2^ > 0.99). In contrast, when submitted to chemical analysis within 48 hr of necropsy, repeatability between two different ∼0.10 g samples from the same liver was somewhat lower (r^2^ = 0.77, n = 16).

Although the experiment was designed to obtain 64 sex-diet-strain cohorts each containing eight mice, breeding success varied among strains, resulting in unequal numbers of animals in each cohort. The average number of mice in each sex-diet-strain cohort was seven. A total of 479 mice, which included 425 RI strain, 25 LG/J, and 29 SM/J mice, were analyzed in this study (supporting information, Table S1 and Table S2).

DNA was extracted from livers using QIAGEN DNeasy Blood and Tissue Kits. All RI strains, SM/J, and LG/J were genotyped at 1436 SNP loci using Illumina Golden Gate technology, 512 of which were nonredundant and informative (Table S3). These loci were distributed as evenly as possible throughout the genome, on all 19 autosomes and the X chromosome. Bonferroni-adjusted chromosome-wide and genome-wide 5% significance thresholds for QTL analysis were calculated using the method of Li and Ji (2005).

### Statistical analysis

The mass of liver analyzed for fat and lean content was not standardized between samples and, of course, different animals have different-sized livers. Thus, statistical analysis, including QTL analysis, was performed on the residuals of liver fat mass regressed onto lean mass (“liver fattiness”), except where noted.

A three-way mixed model analysis of variance with fixed-effects sex and diet and random effect strain was used to calculate variance and broad-sense heritability:

$${\text{Y}}_{\mathrm{ijk}}=\text{μ}+\text{Se}{\text{x}}_{i}+\text{Die}{\text{t}}_{j}+\text{Strai}{\text{n}}_{k}+\left(\text{Se}{\text{x}}_{i}\times \text{Die}{\text{t}}_{j}\right)+\left(\text{Se}{\text{x}}_{i}\times \text{Strai}{\text{n}}_{k}\right)+\left(\text{Die}{\text{t}}_{j}\times \text{Strai}{\text{n}}_{k}\right)+\left(\text{Se}{\text{x}}_{i}\times \text{Die}{\text{t}}_{j}\times \text{Strai}{\text{n}}_{k}\right)+{\text{e}}_{ijk}$$

Variance caused by the strain (σ^2^_str_) represents the total genetic variance among individuals:

$${\text{σ}}_{\text{str}}^{2}=(\text{M}{\text{S}}_{\text{str}}-\text{M}S_{r})/n$$

where MS is the mean square, r is the residual, and *n* is the total number of animals per strain. An adjusted *n* was used to account for the variation in sample size (see *Materials and Methods* section) according to the method of Sokal and Rohlf (1995). Variance caused by the two-way interactions of sex and strain and diet and strain and the three-way interaction of sex, diet, and strain also were calculated using the following formulas, respectively:

$${\text{σ}}_{\text{sexstr}}^{2}=(\text{M}{\text{S}}_{\text{sexstr}}-\text{M}S_{r})/n$$

$${\text{σ}}_{\text{dietstr}}^{2}=(\text{M}{\text{S}}_{\text{dietstr}}-\text{M}S_{r})/n$$

$${\text{σ}}_{\text{sexdietstr}}^{2}=(\text{M}{\text{S}}_{\text{sexdietstr}}-\text{M}S_{r})/n$$

where *n* is to the number of animals per sex-strain, diet-strain, or sex-diet-strain cohort. Broad-sense heritabilities (H^2^), the ratio of genetic to phenotypic variance, were calculated for liver fattiness overall and for each sex-diet cohort:

$${\text{H}}^{2}={\text{σ}}_{\text{str}}^{2}/\left({\text{σ}}_{\text{str}}^{2}+{\text{σ}}_{\text{r}}^{2}\right)$$

Genetic correlations (between-strain correlations) between liver fattiness and other dietary obesity traits in this population (Cheverud *et al.* 2004b) were determined using Pearson product moment correlation.

Mean liver fattiness was calculated for each strain-sex-diet cohort, and these values were used as the individual trait values in QTL analysis (n = 72). The mean can be used as all individuals within a strain are genetically identical. QTL analysis was performed using a maximum likelihood approach with the mixed model procedure in SAS 9.2 (SAS Institute, Cary, NC). The model includes the effects of sex, diet, genotype, and their interactions:

$${\text{Y}}_{ijkl}=\text{μ}+\text{Se}{\text{x}}_{i}+\text{Die}{\text{t}}_{j}+\text{Genotyp}{\text{e}}_{k}+\left(\text{Se}{\text{x}}_{i}\times \text{Die}{\text{t}}_{j}\right)+\left(\text{Se}{\text{x}}_{i}\times \text{Genotyp}{\text{e}}_{k}\right)+\left(\text{Die}{\text{t}}_{j}\times \text{Genotyp}{\text{e}}_{k}\right)+\left(\text{Se}{\text{x}}_{i}\times \text{Die}{\text{t}}_{j}\times \text{Genotyp}{\text{e}}_{k}\right)+{\text{e}}_{ijkl}$$

Y_*ijkl*_ represents the mean phenotype of a strain-sex-diet cohort of strain *l* with sex *i*, diet *j*, and genotype *k* with constant μ and residual e_*ijkl*_. Sex and diet were fixed effects.

Sex-by-genotype and diet-by-genotype interactions were common, so analyses were also performed on the sex, diet, and sex-diet cohorts separately. QTL were located at SNP positions at which the LOD score (log_10_(1/p)) was greater than the chromosome-wide 5% significance threshold. Ninety-five percent support intervals for each QTL included the one-LOD drop from the peak LOD score by convention.

## Results

**Figure 1:**
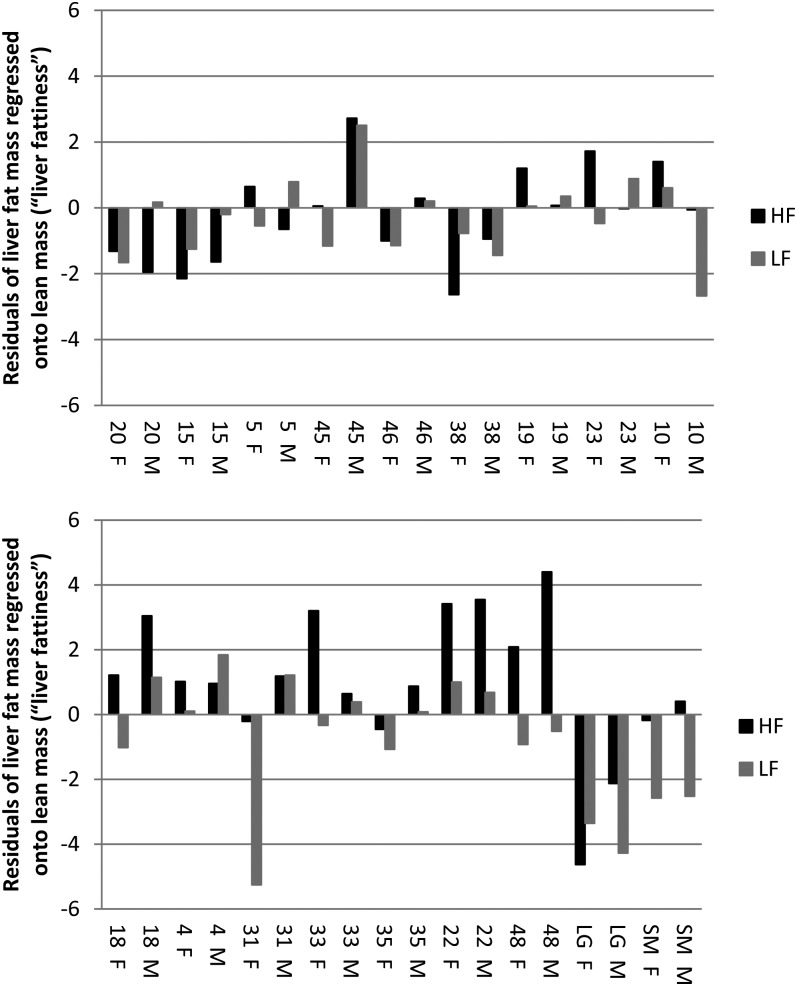
Strain mean residuals of liver fat mass regressed onto lean mass (“liver fattiness”) in male (M) and female (F) mice fed a high-fat (HF) and a low-fat (LF) diet. Numbers and LG, SM represent strains.

The effect of diet on liver fattiness, defined as the residuals of fat mass regressed onto lean mass, was highly significant (*P* = 1.9 × 10^−5^). The effect of sex on liver fattiness was also statistically significant (*P* = 1 × 10^−3^), with females having a fattier liver than males in nine of the 16 RI strains. The effect of strain, the genetic effect, was highly significant for liver fattiness (*P* = 1.3 × 10^−15^) within the population (Figure 1).

A significant strain-by-sex interaction (*P* = 6.7 × 10^−4^) indicated that there was a genetic basis for sexual dimorphism in liver fattiness. The diet-by-sex interaction was also significant (*P* = 0.017) within this population. Most interestingly, a highly significant strain-by-diet interaction (*P* = 8.2 × 10^−6^) supports a genetic basis for variation in liver fattiness in response to dietary fat among the strains. The three-way interaction between sex, diet, and strain, which indicates whether there is a genetic basis for sexual dimorphism in response to dietary fat, was not quite significant (*P* = 0.065).

The genetic correlation between liver fattiness in the high- and low-fat diet environments was 0.61, indicating that 37% of the genetic variance in liver fattiness is shared between the low- and high-fat diets. Thus, most genetic effects on liver fattiness are specific to either the low- or high-fat diet. Genetic correlations between liver fattiness and other dietary obesity- and diabetes-related traits (Cheverud *et al.* 2004b) were also calculated (Table 2). As expected, the genetic correlation between liver fattiness and liver weight (*r* = 0.61) is relatively high. Liver fattiness is also strongly genetically correlated with serum insulin (*r* = 0.63) and serum leptin (*r* = 0.64) levels and moderately correlated with serum cholesterol level (*r* = 0.44). Liver fattiness is negatively correlated with heart (*r* = −0.51) and kidney (*r* = −0.54) weight. Liver fattiness is not significantly correlated with serum free fatty acid levels, serum triglycerides, body weight at necropsy, or any of the four fat pad weights (reproductive, renal, mesenteric, and inguinal).

**Table 2:** Genetic correlations (*r*) between liver fattiness and dietary obesity- and diabetes-related traits (Cheverud *et al.* 2004b)

Obesity-Related Trait	*r*
Leptin, ng/mL	0.64
Insulin, ng/mL	0.63
Liver weight, g	0.61
Cholesterol, mg/dL	0.44
Body weight at necropsy, g	0.28
Mesenteric fat pad weight, g	0.26
Free fatty acids, mmol/L	0
Inguinal fat pad weight, g	−0.01
Total fat pad weight, g	−0.02
Reproductive fat pad weight, g	−0.07
Renal fat pad weight, g	−0.15
Heart weight, g	−0.51
Triglyceride, mg/dL	−0.32
Kidneys weight, g	−0.54

Broad-sense heritability (H^2^) of liver fattiness was 22%. H^2^ within sex-diet cohorts varied from 53% in high-fat fed males to 11% in low-fat fed females. H^2^ in low-fat males and high-fat females was 20% and 31%, respectively. H^2^ of dietary response (strain-by-diet) was 17% and H^2^ of sexual dimorphism (sex-by-strain) was 12%. The three-way sex-diet-strain H^2^ was 9%, indicating that heritable genetic variation for sexual dimorphism in response to dietary fat is limited.

**Figure 2:**
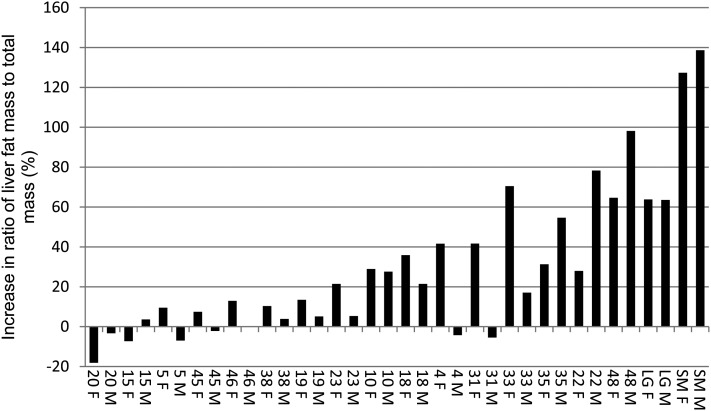
Strain mean percent increase in liver fat content (fat to total mass ratio) in male (M) and female (F) mice fed a high-fat diet over male and female mice fed a low-fat diet. Numbers and LG, SM represent strains.

The aforementioned statistics reveal that the strains differed significantly in the response to the high-fat diet. We characterized the individual strains for liver fat levels and response to a high-fat diet by using percent liver fat rather than liver fattiness for didactic purposes. Among the 16 RI strains, LG/J, and SM/J, the ratio of liver fat mass to total mass significantly (*P* ≤ 0.05) increased in response to a high-fat diet in both males and females in six of the 18 strains (LGXSM-10, 22, 35, 48, SM/J, LG/J; Figure 2). The most extreme liver fat increase from low-fat to high-fat diet was more than120% in the SM/J strain. The RI strain, with the greatest increase in liver fat in response to a high-fat diet, increased 98% in males and 65% in females (LGXSM-48). Liver fat content increased significantly only in females in three strains (LGXSM-4, 18, 33). The livers of eight strains were not significantly responsive to the high-fat diet (LGXSM-5, 15, 19, 20, 23, 38, 45, and 46). The significance of dietary response in LGXSM-31 females was not calculated because this strain included only one low-fat fed female; dietary response is not significant in males of this strain. Mean liver fat mass to total mass ratios, standard deviations, and sample sizes for each sex-diet cohort are listed in Table 3.

**Table 3:** Basic statistics for liver fat content

	All	HF ♀	LF ♀	HF ♂	LF ♂
Average	0.27	0.31	0.24	0.29	0.23
SD	0.08	0.08	0.06	0.1	0.06
N	479	104	127	129	119

Means and SD for liver fat content, defined as the ratio of fat to total mass, for all individuals and sex-diet cohorts individually. N, number of individuals per cohort; HF, high-fat diet; ♀, female; LF, low-fat diet, ♂, male. See Table S1 for statistics of individual strains

### Quantitative trait loci

**Figure 3:**
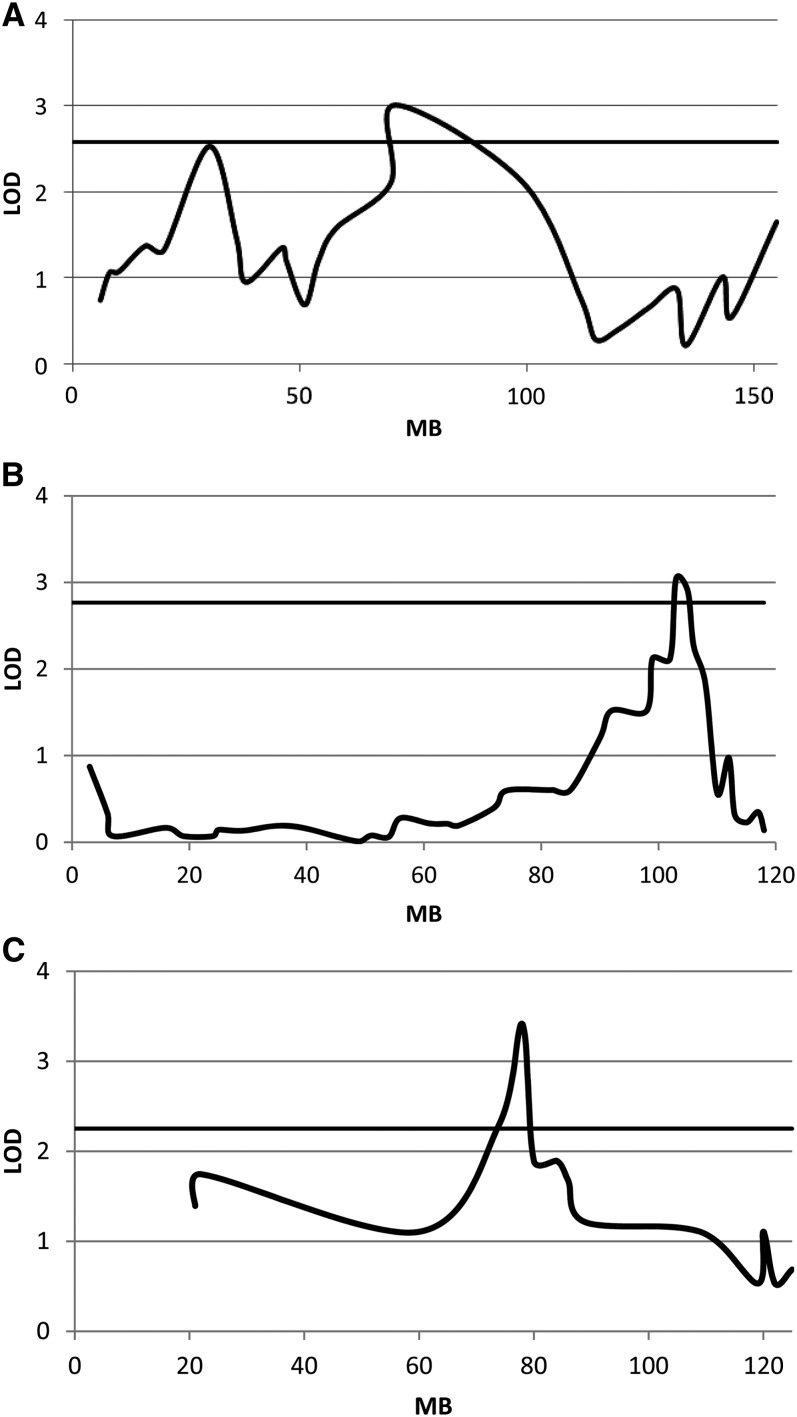
LOD plots of each of the three QTL: (A) *NAFLD4*, (B) *NAFLD11*, and (C) *NAFLD14*. The horizontal line indicates the 5% chromosome-wide significance threshold.

Three QTL that contribute to variation in liver fattiness within the population were identified (Figure 3, Table 4). These QTL were named *NAFLD4*, *NAFLD11*, and *NAFLD14*, with the number indicating their chromosomal location. Genomic regions within these loci contain between 64 and 288 genes. Two of the three QTL were sex-specific, with *NAFLD4* contributing to variation in liver fattiness only in females and *NAFLD14* contributing to variation in liver fattiness only in high fat-fed males.

**Table 4:** QTL for liver fattiness

QTL	SNP	CI	LOD	*a*	No. Genes	Cohort	Other QTL in Interval
*NAFLD4*	rs13477769	70.2-98.5	3.01	1.21	182	Female	Liver weight
*NAFLD11*	rs6180460	98.9-106.2	3.04	0.68	288	All	Liver weight
*NAFLD14*	rs13482262	73.6-80.1	3.41	1.31	64	High Fat Male	Serum glucose, serum insulin

All LOD scores exceed the 5% chromosome-specific thresholds. QTL, quantitative trait loci; SNP, marker at QTL position; CI, confidence interval in MB; *a*, additive genotypic score; no. genes, number of genes in confidence interval. Number in QTL name indicates chromosome number. Other QTL in interval phenotypes from Cheverud *et al.* (2004a) and J. Kenney-Hunt, unpublished data.

Comparison with previously identified dietary obesity and diabetes-related QTL revealed QTL with effects on liver weight in the *NAFLD4* and *NAFLD11* support interval (Cheverud *et al.* 2004a) and for serum insulin and serum glucose within *NAFLD14* (Table 4). An analysis of additive effects was performed to determine whether the LG/J or SM/J allele at each QTL leads to a greater liver fattiness value. The SM/J allele leads to greater liver fattiness than the LG/J allele at all three QTL.

## Discussion

The identification of three QTL that contribute to variation in liver fattiness in the mouse population confirms that the LG/J X SM/J intercross is not only a model for obesity as a complex disease (Ehrich *et al.* 2003; Cheverud *et al.* 2004a,b), but also has potential as a model for the study of NAFLD. Some RI strains respond more strongly to a high-fat diet than others, indicating that these strains could potentially serve as good models of diet-induced NAFLD. Figure 2 provides the percent increase in ratio of liver fat mass to total mass in males and females fed a high-fat diet over males and females fed a low-fat diet by strain. LGXSM-48 responds most strongly to the high-fat diet, with liver fat content in males increasing by 98%. Interestingly, however, although the ratio of liver fat mass to total mass increases by 65% in LGXSM-48 females fed a high-fat diet, mice within this cohort have previously been shown to have insulin and basal glucose levels within the normal range. Males of this strain are hyperinsulemic when fed a high-fat diet (Cheverud *et al.* 2004b).

The RI strains exhibit varying amounts of sexual dimorphism in liver fat in response to the high-fat diet. In LGXSM-33, the ratio of liver fat mass to total mass increased by 70% in females fed a high-fat diet but only by 17% in males. In LGXSM-33 females, basal glucose and insulin levels remained within the normal range, even in females fed a high-fat diet. Males of this strain, however, are both hyperinsulemic and hyperglycemic regardless of their diet and may be considered diabetic (Cheverud *et al.* 2004b). LGXSM-20 and LGXSM-46 are also of interest as strains that fail to increase liver fat on a high-fat diet.

That liver fattiness was not correlated with body weight at necropsy nor the fat depot weights consistent with a study by Fabbrini *et al.* (2009), in which they showed that an increase in visceral adipose tissue is not a good marker for metabolic dysfunction associated with obesity. Fat within the liver correlates with decreased insulin sensitivity and may be a better marker for dysfunction. This finding is consistent with the high correlation between serum insulin and liver fattiness (r^2^ = 0.63).

Because so little is known about the role of genetic variants in NAFLD, this study adds substantial information about NAFLD genetic architecture in addition to the identification of QTL. Because both LG/J and SM/J strains responded with increased liver fattiness on the high-fat diet whereas most of their RI strains responded less extremely, it is possible that dominance and epistasis play an important role in the genetic architecture of this trait. This study cannot address dominance or dominance forms of epistasis by design and contains inadequate strain numbers for an evaluation of epistasis. In the future, liver fat will be quantified in a larger population of F_34_ advanced intercross mice (Wustl:LG,SM-G34; N =1149) for fine mapping QTL. Because the F_34_ population is a randomly mated advanced intercross line (AIL) rather than a RI strain set, the sample size is greatly increased, there are heterozygous individuals in the population, and the population has accumulated more recombination across the genome. These characteristics allow for examination of dominance, epistasis, and for fine-scale (<1 cM) mapping for liver fat phenotypes.

## Supplementary Material

Supporting Information
