# Supplementary material for: Quantitative Trait Loci Affecting Liver Fat Content in Mice
Source: G3 (Bethesda). 2012 Sep 1;2(9):1019–25. doi: 10.1534/g3.112.003343 (PMC3429915; doi:10.1534/g3.112.003343)
Supplement: Supporting Information [file supp_2_9_1019__index.html]

Supporting Information 

# Quantitative Trait Loci Affecting Liver Fat Content in Mice

## Supporting Information for Minkina *et al.*, 2012

**Files in this Data Supplement:**

- Supporting Information - Tables S1-S3 (PDF, 524 KB)
- Table S1 - Number of mice (N), average liver fat content and standard deviation (SD), average liver fattiness and standard deviation (SD), by cohort (PDF, 72 KB)
- Table S2 - Hepatic fat phenotypes (PDF, 136 KB)
- Table S3 - SNP genotypes by strain and chromosome (CHR) (PDF, 409 KB)
