# Supplementary material for: Quantitative Trait Loci Affecting Liver Fat Content in Mice
Source: G3 (Bethesda). 2012 Sep 1;2(9):1019–25. doi: 10.1534/g3.112.003343 (PMC3429915; doi:10.1534/g3.112.003343)
Supplement: Supporting Information [file supp_2.9.1019_003343SI.pdf]

Table S1 **Number of mice (N), average liver fat content and standard deviation (SD), average liver fattiness and standard deviation (SD), by cohort.**

		Liver Fat Content		Liver Fattiness	
**Cohort**	**N**	**Average (%)**	**SD**	**Average (mg)**	**SD**
**4 All**	**21**	**0.30**	**0.04**	**1.02**	**1.39**
4 HF F	3	0.37	0.02	1.02	0.99
4 HF M	5	0.29	0.03	0.96	0.89
4 LF F	6	0.26	0.02	0.10	1.23
4 LF M	7	0.30	0.04	1.84	1.63
**5 All**	**28**	**0.26**	**0.05**	**-0.04**	**1.77**
5 HF F	7	0.29	0.04	0.65	1.67
5 HF M	9	0.24	0.04	-0.65	1.48
5 LF F	7	0.27	0.05	-0.55	2.21
5 LF M	5	0.26	0.03	0.79	1.55
**10 All**	**26**	**0.25**	**0.06**	**-0.43**	**2.53**
10 HF F	5	0.34	0.03	1.40	1.96
10 HF M	7	0.25	0.05	-0.06	2.21
10 LF F	6	0.26	0.02	0.61	0.75
10 LF M	8	0.19	0.04	-2.68	2.60
**15 All**	**31**	**0.22**	**0.05**	**-1.27**	**1.94**
15 HF F	6	0.23	0.05	-2.15	2.75
15 HF M	9	0.21	0.05	-1.64	2.19
15 LF F	9	0.24	0.04	-1.25	1.40
15 LF M	7	0.20	0.02	-0.20	1.09
**18 All**	**28**	**0.29**	**0.07**	**0.94**	**2.18**
18 HF F	6	0.31	0.03	1.22	0.99
18 HF M	7	0.35	0.07	3.05	1.76
18 LF F	9	0.23	0.03	-1.02	1.28
18 LF M	6	0.29	0.08	1.15	2.23
**19 All**	**31**	**0.26**	**0.04**	**0.41**	**1.54**
19HF F	7	0.30	0.05	1.20	0.40
19 HF M	8	0.26	0.02	0.07	1.62
19 LF F	7	0.26	0.04	0.05	1.12
19 LF M	9	0.24	0.05	0.35	2.19

**20 All**	**16**	**0.25**	**0.13**	**-1.12**	**2.50**
20 HF F	2	0.22	0.00	-1.32	0.89
20 HF M	4	0.24	0.19	-1.95	1.55
20 LF F	5	0.26	0.14	-1.66	2.10
20 LF M	5	0.25	0.12	0.17	3.74
**22 All**	**44**	**0.37**	**0.10**	**2.30**	**2.44**
22 HF F	12	0.41	0.06	3.42	1.82
22 HF M	12	0.46	0.05	3.55	2.25
22 LF F	12	0.32	0.06	1.00	2.63
22 LF M	8	0.26	0.08	0.69	1.38
**23 All**	**38**	**0.26**	**0.06**	**0.48**	**2.03**
23 HF F	7	0.31	0.07	1.72	2.71
23 HF M	10	0.25	0.06	-0.02	1.50
23 LF F	9	0.25	0.05	-0.48	1.73
23 LF M	12	0.24	0.04	0.89	1.94
**31 All**	**11**	**0.26**	**0.04**	**0.19**	**2.34**
31 HF F	4	0.27	0.05	-0.21	2.17
31 HF M	5	0.26	0.05	1.19	1.37
31 LF F	1	0.19	--	-5.26	--
31 LF M	2	0.28	0.01	1.22	1.04
**33 All**	**23**	**0.28**	**0.08**	**0.74**	**1.79**
33 HF F	4	0.40	0.09	3.20	0.83
33 HF M	7	0.28	0.05	0.65	1.20
33 LF F	7	0.23	0.03	-0.33	0.98
33 LF M	5	0.23	0.02	0.39	2.27
**35 All**	**26**	**0.25**	**0.07**	**-0.16**	**1.60**
35 HF F	6	0.26	0.04	-0.45	1.50
35 HF M	5	0.36	0.09	0.88	2.12
35 LF F	7	0.20	0.03	-1.07	1.34
35 LF M	8	0.23	0.04	0.08	1.11

**38 All**	**30**	**0.21**	**0.05**	**-1.43**	**1.95**
38 HF F	7	0.25	0.04	-2.64	3.15
38 HF M	7	0.18	0.04	-0.95	1.33
38 LF F	8	0.23	0.04	-0.78	1.21
38 LF M	8	0.17	0.03	-1.44	1.45
**45 All**	**16**	**0.29**	**0.03**	**0.64**	**2.85**
45 HF F	5	0.29	0.03	0.05	3.60
45 HF M	3	0.31	0.02	2.72	0.67
45 LF F	5	0.27	0.04	-1.16	2.58
45 LF M	3	0.32	0.02	2.51	0.72
**46 All**	**27**	**0.23**	**0.06**	**-0.40**	**2.14**
46 HF F	5	0.23	0.09	-1.00	3.73
46 HF M	6	0.24	0.07	0.29	1.18
46 LF F	8	0.21	0.04	-1.15	1.59
46 LF M	8	0.24	0.05	0.21	1.96
**48 All**	**28**	**0.31**	**0.11**	**1.14**	**3.19**
48 HF F	6	0.37	0.10	2.09	4.15
48 HF M	7	0.43	0.05	4.40	1.41
48 LF F	9	0.22	0.06	-0.92	2.02
48 LF M	6	0.22	0.05	-0.52	1.38
**LG All**	**25**	**0.19**	**0.05**	**-3.60**	**2.97**
LG HF F	6	0.24	0.02	-4.63	3.32
LG HF M	7	0.22	0.03	-2.13	2.32
LG LF F	6	0.15	0.03	-3.36	4.09
LG LF M	6	0.13	0.02	-4.27	1.74
**SM All**	**29**	**0.25**	**0.11**	**-0.94**	**2.48**
SM HF F	6	0.33	0.05	-0.18	1.97
SM HF M	11	0.33	0.10	0.41	2.51
SM LF F	6	0.14	0.02	-2.58	2.20
SM LF M	6	0.14	0.02	-2.52	1.50

Table S2 Hepatic fat phenotypes.

	Sex	Diet	Strain	Fattiness	Fat Content
1	♀	LF	SM/J	-4.89	0.17
2	♀	LF	SM/J	-4.76	0.12
3	♂	LF	SM/J	-4.48	0.11
4	♂	LF	SM/J	-3.87	0.11
5	♂	HF	SM/J	-3.86	0.16
6	♀	LF	SM/J	-3.34	0.14
7	♂	LF	SM/J	-2.55	0.13
8	♀	LF	SM/J	-2.50	0.16
9	♀	HF	SM/J	-2.33	0.32
10	♂	LF	SM/J	-2.02	0.17
11	♂	LF	SM/J	-1.91	0.15
12	♀	HF	SM/J	-1.55	0.31
13	♂	HF	SM/J	-1.52	0.25
14	♀	HF	SM/J	-1.37	0.29
15	♂	HF	SM/J	-0.86	0.29
16	♂	HF	SM/J	-0.83	0.15
17	♂	HF	SM/J	-0.54	0.37
18	♀	LF	SM/J	-0.29	0.14
19	♂	LF	SM/J	-0.29	0.15
20	♀	HF	SM/J	-0.03	0.28
21	♂	HF	SM/J	0.15	0.41
22	♀	LF	SM/J	0.31	0.14
23	♂	HF	SM/J	0.85	0.34
24	♂	HF	SM/J	0.87	0.45
25	♂	HF	SM/J	0.97	0.37
26	♀	HF	SM/J	1.39	0.39
27	♀	HF	SM/J	2.81	0.37
28	♂	HF	SM/J	4.20	0.43
29	♂	HF	SM/J	5.06	0.40
30	♀	LF	LG/J	-9.99	0.14
31	♀	HF	LG/J	-8.94	0.24
32	♀	HF	LG/J	-7.72	0.23
33	♂	LF	LG/J	-6.71	0.15
34	♀	LF	LG/J	-6.05	0.12
35	♀	HF	LG/J	-5.33	0.23
36	♂	LF	LG/J	-5.26	0.12
37	♂	LF	LG/J	-5.21	0.11
38	♂	HF	LG/J	-4.81	0.25

39	♂	HF	LG/J	-4.31	0.17
40	♀	LF	LG/J	-3.82	0.12
41	♂	LF	LG/J	-3.61	0.14
42	♀	HF	LG/J	-3.50	0.27
43	♂	HF	LG/J	-3.03	0.20
44	♂	LF	LG/J	-2.57	0.16
45	♂	LF	LG/J	-2.28	0.13
46	♀	HF	LG/J	-1.86	0.26
47	♂	HF	LG/J	-1.19	0.21
48	♂	HF	LG/J	-0.57	0.23
49	♀	HF	LG/J	-0.42	0.23
50	♀	LF	LG/J	-0.17	0.16
51	♀	LF	LG/J	-0.11	0.19
52	♀	LF	LG/J	0.01	0.16
53	♂	HF	LG/J	1.15	0.22
54	♂	LF	4	-1.31	0.24
55	♀	LF	4	-1.27	0.23
56	♀	LF	4	-1.13	0.25
57	♀	LF	4	0.02	0.29
58	♂	HF	4	0.12	0.26
59	♂	HF	4	0.12	0.27
60	♀	LF	4	0.13	0.26
61	♀	HF	4	0.16	0.35
62	♀	HF	4	0.79	0.37
63	♂	HF	4	0.87	0.27
64	♀	LF	4	0.92	0.28
65	♂	LF	4	1.33	0.33
66	♂	HF	4	1.51	0.33
67	♂	LF	4	1.81	0.33
68	♂	LF	4	1.84	0.35
69	♀	LF	4	1.97	0.27
70	♀	HF	4	2.11	0.40
71	♂	HF	4	2.16	0.32
72	♂	LF	4	2.29	0.28
73	♂	LF	4	3.21	0.29
74	♂	LF	4	3.75	0.29
75	♀	LF	5	-3.73	0.25
76	♂	HF	5	-2.64	0.22
77	♂	HF	5	-2.43	0.28
78	♀	LF	5	-1.79	0.23
79	♀	LF	5	-1.79	0.24
80	♂	HF	5	-1.37	0.26

81	♀	HF	5	-1.36	0.22
82	♂	LF	5	-1.01	0.22
83	♀	HF	5	-0.94	0.27
84	♂	HF	5	-0.93	0.20
85	♂	HF	5	-0.92	0.23
86	♀	HF	5	-0.86	0.27
87	♀	LF	5	-0.46	0.32
88	♂	LF	5	-0.42	0.30
89	♂	HF	5	-0.39	0.19
90	♂	HF	5	-0.14	0.20
91	♀	LF	5	-0.10	0.23
92	♂	LF	5	0.77	0.23
93	♀	LF	5	0.86	0.25
94	♀	HF	5	0.93	0.28
95	♂	HF	5	1.18	0.30
96	♂	HF	5	1.79	0.28
97	♂	LF	5	1.99	0.24
98	♀	HF	5	2.01	0.33
99	♀	HF	5	2.27	0.33
100	♀	HF	5	2.47	0.34
101	♂	LF	5	2.63	0.29
102	♀	LF	5	3.18	0.35
103	♂	LF	10	-6.86	0.16
104	♂	LF	10	-5.55	0.18
105	♂	LF	10	-3.55	0.15
106	♂	HF	10	-3.15	0.28
107	♂	LF	10	-2.87	0.20
108	♂	LF	10	-2.28	0.18
109	♂	HF	10	-1.41	0.17
110	♂	HF	10	-0.85	0.20
111	♂	HF	10	-0.84	0.25
112	♀	HF	10	-0.64	0.35
113	♂	LF	10	-0.52	0.26
114	♂	LF	10	-0.34	0.24
115	♀	LF	10	-0.06	0.25
116	♀	LF	10	0.03	0.24
117	♀	HF	10	0.21	0.38
118	♀	LF	10	0.37	0.26
119	♀	LF	10	0.39	0.30
120	♀	HF	10	0.44	0.30
121	♂	HF	10	0.50	0.24
122	♂	LF	10	0.57	0.18

123	♀	LF	10	0.99	0.27
124	♂	HF	10	1.74	0.28
125	♀	LF	10	1.94	0.27
126	♀	HF	10	3.48	0.33
127	♀	HF	10	3.54	0.35
128	♂	HF	10	3.58	0.32
129	♀	HF	15	-5.93	0.14
130	♂	HF	15	-5.72	0.15
131	♀	HF	15	-4.23	0.26
132	♀	LF	15	-3.77	0.18
133	♂	HF	15	-3.35	0.15
134	♀	HF	15	-3.22	0.18
135	♂	HF	15	-3.21	0.29
136	♀	LF	15	-2.86	0.30
137	♂	LF	15	-2.78	0.23
138	♂	HF	15	-2.01	0.15
139	♀	LF	15	-1.31	0.29
140	♂	HF	15	-1.26	0.17
141	♀	LF	15	-1.07	0.20
142	♀	HF	15	-0.97	0.26
143	♂	HF	15	-0.88	0.19
144	♀	LF	15	-0.88	0.23
145	♀	LF	15	-0.40	0.21
146	♂	LF	15	-0.29	0.19
147	♂	HF	15	-0.29	0.26
148	♂	LF	15	-0.23	0.18
149	♀	LF	15	0.03	0.24
150	♂	LF	15	0.13	0.20
151	♂	LF	15	0.18	0.21
152	♀	LF	15	0.22	0.25
153	♂	LF	15	0.28	0.20
154	♂	LF	15	0.38	0.19
155	♀	HF	15	0.51	0.26
156	♂	LF	15	0.71	0.28
157	♂	HF	15	0.74	0.25
158	♀	HF	15	0.93	0.26
159	♂	HF	15	1.21	0.26
160	♀	LF	18	-2.91	0.26
161	♀	LF	18	-2.54	0.20
162	♂	LF	18	-2.17	0.26
163	♀	LF	18	-1.85	0.22
164	♀	LF	18	-1.21	0.20

165	♀	LF	18	-1.10	0.25
166	♀	LF	18	-0.87	0.21
167	♀	LF	18	0.04	0.18
168	♀	HF	18	0.18	0.31
169	♀	HF	18	0.30	0.29
170	♂	HF	18	0.38	0.24
171	♂	LF	18	0.50	0.28
172	♀	HF	18	0.52	0.30
173	♀	LF	18	0.56	0.27
174	♀	LF	18	0.68	0.26
175	♂	LF	18	0.84	0.25
176	♂	LF	18	0.95	0.25
177	♂	HF	18	1.81	0.38
178	♀	HF	18	1.88	0.28
179	♀	HF	18	1.99	0.32
180	♂	LF	18	2.11	0.28
181	♀	HF	18	2.43	0.35
182	♂	HF	18	2.53	0.27
183	♂	HF	18	2.81	0.35
184	♂	HF	18	3.93	0.41
185	♂	HF	18	4.04	0.37
186	♂	LF	18	4.66	0.45
187	♂	HF	18	5.84	0.46
188	♂	HF	19	-3.57	0.23
189	♂	LF	19	-3.02	0.20
190	♀	LF	19	-1.75	0.25
191	♂	LF	19	-1.73	0.24
192	♂	LF	19	-1.37	0.21
193	♂	HF	19	-0.60	0.22
194	♀	LF	19	-0.56	0.23
195	♀	LF	19	-0.40	0.23
196	♀	LF	19	-0.16	0.28
197	♂	LF	19	-0.14	0.17
198	♂	LF	19	0.05	0.23
199	♂	HF	19	0.42	0.24
200	♂	HF	19	0.43	0.25
201	♂	HF	19	0.45	0.26
202	♀	HF	19	0.74	0.28
203	♂	HF	19	0.75	0.27
204	♀	LF	19	0.75	0.23
205	♀	HF	19	0.76	0.30
206	♂	HF	19	0.82	0.30

207	♀	LF	19	0.83	0.29
208	♀	HF	19	0.99	0.31
209	♂	LF	19	1.24	0.29
210	♀	HF	19	1.27	0.28
211	♀	HF	19	1.36	0.36
212	♀	HF	19	1.49	0.21
213	♀	LF	19	1.64	0.33
214	♀	HF	19	1.82	0.34
215	♂	HF	19	1.89	0.27
216	♂	LF	19	2.07	0.24
217	♂	LF	19	2.27	0.31
218	♂	LF	19	3.83	0.29
219	♀	LF	20	-4.30	0.18
220	♂	HF	20	-4.26	0.52
221	♂	LF	20	-3.70	0.18
222	♀	LF	20	-3.41	0.16
223	♂	LF	20	-3.27	0.15
224	♀	HF	20	-1.95	0.22
225	♂	HF	20	-1.43	0.15
226	♂	HF	20	-1.14	0.13
227	♂	HF	20	-0.99	0.15
228	♀	LF	20	-0.93	0.14
229	♀	HF	20	-0.69	0.22
230	♀	LF	20	-0.30	0.39
231	♂	LF	20	0.49	0.17
232	♀	LF	20	0.63	0.45
233	♂	LF	20	2.16	0.32
234	♂	LF	20	5.17	0.42
235	♀	LF	22	-4.83	0.32
236	♀	LF	22	-2.18	0.24
237	♂	LF	22	-2.00	0.12
238	♂	HF	22	-0.77	0.47
239	♀	LF	22	-0.74	0.28
240	♂	LF	22	-0.37	0.22
241	♀	HF	22	-0.25	0.28
242	♀	LF	22	-0.16	0.25
243	♂	HF	22	0.12	0.45
244	♀	LF	22	0.28	0.38
245	♂	LF	22	0.36	0.40
246	♂	LF	22	0.61	0.22
247	♀	HF	22	0.92	0.47
248	♂	LF	22	1.31	0.28

249	♂	LF	22	1.43	0.27
250	♀	LF	22	1.60	0.27
251	♀	HF	22	1.67	0.45
252	♂	LF	22	1.85	0.26
253	♂	HF	22	2.04	0.50
254	♀	LF	22	2.21	0.31
255	♂	LF	22	2.30	0.32
256	♀	LF	22	2.46	0.30
257	♀	LF	22	2.57	0.35
258	♂	HF	22	2.88	0.43
259	♀	HF	22	2.96	0.35
260	♂	HF	22	3.16	0.50
261	♀	LF	22	3.21	0.36
262	♀	HF	22	3.39	0.46
263	♂	HF	22	3.41	0.48
264	♀	HF	22	3.62	0.37
265	♂	HF	22	3.65	0.34
266	♀	LF	22	3.80	0.38
267	♀	LF	22	3.81	0.42
268	♀	HF	22	3.99	0.39
269	♀	HF	22	4.18	0.42
270	♀	HF	22	4.77	0.44
271	♀	HF	22	4.78	0.41
272	♂	HF	22	4.93	0.48
273	♂	HF	22	5.39	0.49
274	♀	HF	22	5.47	0.44
275	♀	HF	22	5.52	0.45
276	♂	HF	22	5.54	0.44
277	♂	HF	22	6.00	0.49
278	♂	HF	22	6.23	0.50
279	♀	HF	23	-2.82	0.27
280	♂	LF	23	-2.57	0.14
281	♂	HF	23	-2.48	0.25
282	♂	HF	23	-2.32	0.20
283	♀	LF	23	-2.10	0.19
284	♀	LF	23	-1.91	0.23
285	♀	LF	23	-1.86	0.32
286	♀	LF	23	-1.79	0.25
287	♀	LF	23	-1.63	0.17
288	♀	HF	23	-1.29	0.16
289	♂	LF	23	-0.92	0.20
290	♂	LF	23	-0.82	0.22

291	♂	LF	23	-0.51	0.22
292	♂	HF	23	-0.43	0.20
293	♂	HF	23	-0.27	0.17
294	♂	HF	23	0.11	0.28
295	♂	LF	23	0.13	0.23
296	♂	HF	23	0.18	0.37
297	♀	LF	23	0.58	0.28
298	♂	HF	23	0.61	0.25
299	♂	HF	23	0.71	0.20
300	♀	LF	23	0.79	0.28
301	♂	LF	23	0.82	0.24
302	♂	LF	23	0.84	0.25
303	♀	LF	23	1.13	0.28
304	♂	HF	23	1.17	0.28
305	♂	LF	23	1.46	0.28
306	♀	HF	23	2.00	0.30
307	♂	LF	23	2.43	0.28
308	♂	LF	23	2.45	0.24
309	♂	HF	23	2.46	0.31
310	♀	LF	23	2.52	0.27
311	♀	HF	23	2.70	0.32
312	♂	LF	23	3.46	0.29
313	♀	HF	23	3.53	0.36
314	♂	LF	23	3.88	0.28
315	♀	HF	23	3.91	0.37
316	♀	HF	23	4.03	0.36
317	♀	LF	31	-5.26	0.19
318	♀	HF	31	-3.34	0.31
319	♂	HF	31	-0.59	0.21
320	♀	HF	31	0.05	0.22
321	♂	LF	31	0.48	0.29
322	♂	HF	31	1.02	0.32
323	♂	HF	31	1.03	0.24
324	♀	HF	31	1.04	0.32
325	♂	HF	31	1.21	0.24
326	♀	HF	31	1.42	0.25
327	♂	LF	31	1.96	0.27
328	♂	HF	31	3.27	0.29
329	♂	LF	33	-2.79	0.21
330	♀	LF	33	-2.35	0.18
331	♂	HF	33	-1.71	0.18
332	♂	LF	33	-0.45	0.23

333	♀	LF	33	-0.34	0.25
334	♀	LF	33	-0.26	0.24
335	♀	LF	33	-0.23	0.25
336	♀	LF	33	-0.13	0.25
337	♀	LF	33	0.11	0.20
338	♂	LF	33	0.12	0.22
339	♂	HF	33	0.14	0.34
340	♂	HF	33	0.35	0.27
341	♀	LF	33	0.85	0.27
342	♂	HF	33	1.15	0.30
343	♂	HF	33	1.18	0.28
344	♂	HF	33	1.64	0.30
345	♂	HF	33	1.77	0.25
346	♂	LF	33	2.04	0.25
347	♀	HF	33	2.19	0.29
348	♀	HF	33	2.87	0.39
349	♂	LF	33	3.04	0.27
350	♀	HF	33	3.72	0.52
351	♀	HF	33	4.02	0.40
352	♀	LF	35	-3.02	0.16
353	♀	LF	35	-2.36	0.17
354	♂	LF	35	-2.26	0.29
355	♂	HF	35	-2.13	0.26
356	♀	HF	35	-2.02	0.26
357	♀	LF	35	-1.95	0.21
358	♀	HF	35	-1.41	0.27
359	♀	HF	35	-1.25	0.28
360	♀	HF	35	-0.70	0.19
361	♂	LF	35	-0.46	0.18
362	♂	HF	35	-0.45	0.46
363	♀	LF	35	-0.43	0.19
364	♀	LF	35	-0.21	0.22
365	♂	LF	35	0.03	0.21
366	♀	LF	35	0.05	0.22
367	♂	LF	35	0.06	0.19
368	♀	LF	35	0.41	0.22
369	♂	LF	35	0.43	0.23
370	♂	LF	35	0.51	0.24
371	♂	HF	35	0.70	0.44
372	♀	HF	35	0.73	0.30
373	♂	HF	35	0.88	0.27
374	♂	LF	35	1.01	0.23

375	♂	LF	35	1.34	0.28
376	♀	HF	35	1.94	0.27
377	♂	HF	35	2.26	0.26
378	♂	HF	35	3.99	0.36
379	♀	HF	38	-6.51	0.20
380	♀	HF	38	-5.45	0.25
381	♀	HF	38	-5.26	0.24
382	♂	HF	38	-3.57	0.13
383	♂	LF	38	-3.28	0.21
384	♂	LF	38	-3.18	0.15
385	♀	LF	38	-2.69	0.22
386	♀	HF	38	-2.67	0.23
387	♂	LF	38	-2.29	0.14
388	♀	LF	38	-2.00	0.20
389	♂	LF	38	-1.65	0.18
390	♂	HF	38	-1.28	0.13
391	♂	LF	38	-1.24	0.21
392	♂	HF	38	-1.03	0.16
393	♀	LF	38	-0.95	0.18
394	♀	LF	38	-0.76	0.30
395	♀	LF	38	-0.74	0.22
396	♂	HF	38	-0.64	0.21
397	♀	LF	38	-0.61	0.21
398	♂	HF	38	-0.51	0.21
399	♂	LF	38	-0.51	0.14
400	♂	HF	38	-0.41	0.22
401	♂	LF	38	-0.24	0.18
402	♀	HF	38	0.00	0.26
403	♀	LF	38	0.44	0.25
404	♀	HF	38	0.48	0.28
405	♂	HF	38	0.81	0.20
406	♂	LF	38	0.83	0.17
407	♀	HF	38	0.95	0.33
408	♀	LF	38	1.11	0.27
409	♀	LF	45	-5.40	0.24
410	♀	HF	45	-5.32	0.26
411	♀	HF	45	-1.70	0.29
412	♀	LF	45	-1.20	0.22
413	♀	LF	45	-0.41	0.28
414	♀	LF	45	-0.31	0.28
415	♀	HF	45	1.53	0.30
416	♀	LF	45	1.54	0.33

417	♂	HF	45	1.97	0.32
418	♀	HF	45	1.98	0.27
419	♂	LF	45	2.05	0.29
420	♂	LF	45	2.13	0.34
421	♂	HF	45	2.92	0.31
422	♂	HF	45	3.27	0.29
423	♂	LF	45	3.34	0.32
424	♀	HF	45	3.79	0.34
425	♀	HF	46	-6.92	0.19
426	♂	LF	46	-4.20	0.17
427	♀	LF	46	-3.88	0.16
428	♀	LF	46	-2.90	0.13
429	♀	LF	46	-2.04	0.21
430	♀	HF	46	-1.53	0.14
431	♂	HF	46	-1.33	0.18
432	♂	HF	46	-0.57	0.27
433	♀	LF	46	-0.56	0.19
434	♂	LF	46	-0.44	0.17
435	♀	LF	46	-0.32	0.25
436	♀	HF	46	-0.29	0.19
437	♂	HF	46	-0.28	0.18
438	♀	LF	46	0.02	0.22
439	♀	LF	46	0.05	0.26
440	♂	LF	46	0.09	0.19
441	♂	LF	46	0.17	0.23
442	♀	LF	46	0.46	0.23
443	♀	HF	46	0.56	0.31
444	♂	HF	46	0.93	0.17
445	♂	LF	46	1.28	0.30
446	♂	HF	46	1.44	0.29
447	♂	LF	46	1.54	0.26
448	♂	HF	46	1.55	0.33
449	♂	LF	46	1.55	0.28
450	♂	LF	46	1.68	0.28
451	♀	HF	46	3.17	0.34
452	♀	LF	48	-5.10	0.16
453	♀	HF	48	-4.85	0.20
454	♀	LF	48	-3.12	0.17
455	♂	LF	48	-2.75	0.16
456	♀	LF	48	-1.33	0.16
457	♀	LF	48	-1.17	0.18
458	♂	LF	48	-1.04	0.18

459	♂	LF	48	-0.65	0.25
460	♂	LF	48	-0.43	0.20
461	♀	HF	48	-0.08	0.31
462	♀	LF	48	0.04	0.25
463	♀	LF	48	0.50	0.29
464	♂	LF	48	0.53	0.21
465	♀	LF	48	0.55	0.26
466	♀	LF	48	0.64	0.23
467	♀	LF	48	0.66	0.31
468	♂	LF	48	1.24	0.30
469	♀	HF	48	2.12	0.39
470	♂	HF	48	2.97	0.35
471	♀	HF	48	3.17	0.41
472	♂	HF	48	3.43	0.40
473	♂	HF	48	3.57	0.45
474	♂	HF	48	3.98	0.46
475	♂	HF	48	4.14	0.41
476	♂	HF	48	5.94	0.46
477	♀	HF	48	5.95	0.43
478	♀	HF	48	6.23	0.48
479	♂	HF	48	6.78	0.50

♀ = female, ♂ = male, HF = high fat diet, LF = low fat diet, Fattiness = residuals of liver fat mass regressed onto lean mass, Fat Content = ratio of liver fat mass to total liver mass. Strain numbers are prefaced by LGXSM-, except strains Large (LG/J) and Small (SM/J).

Table S3   SNP genotypes by strain and chromosome (CHR).

CHR	SNP	SM/J	LG/J	4	5	10	15	18	19	20
1	rs3683945	SS	LL	LL	SS	LL	LL	LL	SS	LL
1	rs13475748	SS	LL	LL	SS	LL	LL	LL	LL	LL
1	rs13475774	SS	LL	LL	LL	LL	LL	LL	LL	LL
1	rs13475816	SS	LL	LL	LL	LL	LL	LL	LL	LL
1	rs3685569	SS	LL	LL	LL	LL	LL	LL	LL	LL
1	gnf01.037.906	SS	LL	LL	LL	SS	LL	LL	LL	LL
1	rs3663706	SS	LL	LL	SS	SS	LL	LL	LL	LL
1	rs13475837	SS	LL	LL	LL	SS	LL	LL	LL	LL
1	rs13475863	SS	LL	LL	LL	SS	LL	LL	LL	LL
1	rs13475874	SS	LL	LL	LL	SS	LL	LL	LL	LL
1	petAF067836-350A-1	SS	LL	LL	LL	SS	LL	LL	LL	LL
1	rs13475894	SS	LL	LL	LL	SS	LL	LL	LL	LL
1	rs13475902	SS	LL	LL	LL	LL	LL	LL	LL	LL
1	rs13475903	SS	LL	LL	LL	LL	LL	LL	LL	LL
1	rs13475931	SS	LL	LL	LL	LL	LL	LL	LL	LL
1	rs3667200	SS	LL	LL	LL	LL	LL	LL	LL	LL
1	rs13475970	SS	LL	LL	LL	SS	LL	LL	LL	LL
1	rs3664800	SS	LL	LL	LL	SS	LL	LL	LL	SS
1	rs6342650	SS	LL	LL	LL	LL	LL	LL	LL	SS
1	rs13476036	SS	LL	LL	LL	LL	LL	LL	LL	SS
1	mCV22824651	SS	LL	LL	LL	LL	LL	SS	LL	SS
1	rs3719973	SS	LL	LL	LL	LL	LL	SS	LL	SS
1	rs3686671	SS	LL	LL	LL	LL	LL	LL	LL	LL
1	rs3691374	SS	LL	LL	LL	LL	LL	LL	LL	LL
1	rs13476122	SS	LL	LL	SS	LL	LL	LL	LL	LL
1	rs13476125	SS	LL	NA	SS	LL	LL	LL	LL	LL
1	rs6250257	SS	LL	NA	SS	LL	LL	LL	SS	LL
1	rs13476136	SS	LL	NA	SS	LL	LL	LL	SS	LL
1	rs6186115	SS	LL	NA	SS	LL	LL	LL	SS	LL
1	gnf01.157.188	SS	LL	LL	SS	LL	LL	SS	SS	LL
1	rs13476211	SS	LL	LL	SS	LL	LL	SS	SS	SS
1	rs8256589	SS	LL	LL	SS	LL	LL	SS	SS	SS
1	rs8242852	SS	LL	LL	SS	LL	LL	SS	SS	LL
1	rs13476248	SS	LL	LL	SS	LL	LL	SS	SS	LL
1	rs3723788	SS	LL	LL	LL	LL	LL	SS	SS	LL
1	mCV23509126	SS	LL	LL	SS	LL	LL	SS	SS	LL
1	rs6157620	SS	LL	LL	SS	LL	LL	SS	SS	LL
1	rs13476286	SS	LL	LL	SS	LL	LL	SS	SS	SS

1	rs13476293	SS	LL	SS	LL	LL	LL	SS	SS	SS
1	rs13476308	SS	LL	SS	SS	LL	LL	SS	SS	SS
2	rs13476319	SS	LL	SS	LL	LL	LL	LL	SS	SS
2	rs6359983	SS	LL	SS	LL	LL	LL	LL	SS	SS
2	gnf02.007.482	SS	LL	SS	LL	LL	LL	LL	LL	SS
2	rs3688854	SS	LL	LL	SS	SS	LL	LL	LL	SS
2	mCV23209429	SS	LL	LL	SS	SS	LL	SS	LL	SS
2	rs13476425	SS	LL	LL	SS	SS	LL	SS	LL	SS
2	rs6313371	SS	LL	LL	SS	SS	LL	SS	LL	SS
2	rs3680197	SS	LL	LL	SS	SS	LL	SS	SS	SS
2	rs3718711	SS	LL	LL	SS	SS	LL	SS	LL	SS
2	rs3022886	SS	LL	LL	SS	SS	LL	LL	LL	SS
2	rs8263587	SS	LL	LL	SS	LL	SS	LL	LL	SS
2	rs6295014	SS	LL	LL	SS	LL	LL	LL	SS	SS
2	rs3710094	SS	LL	LL	SS	LL	LL	LL	LL	SS
2	CEL-2_79237503	SS	LL	LL	LL	LL	LL	LL	LL	SS
2	rs13476598	SS	LL	LL	LL	LL	LL	LL	LL	SS
2	rs13476637	SS	LL	LL	LL	LL	LL	LL	LL	SS
2	rs13476647	SS	LL	LL	LL	LL	LL	LL	LL	SS
2	rs6406705	SS	LL	LL	LL	LL	LL	LL	LL	SS
2	rs6208879	SS	LL	LL	LL	LL	LL	LL	LL	SS
2	rs13476723	SS	LL	LL	LL	LL	LL	LL	LL	SS
2	rs3725853	SS	LL	LL	SS	LL	LL	LL	LL	SS
2	rs13476761	SS	LL	LL	SS	LL	LL	LL	LL	SS
2	rs13476787	SS	LL	LL	SS	LL	LL	LL	LL	SS
2	rs6249968	SS	LL	LL	SS	LL	LL	LL	LL	SS
2	mCV23169908	SS	LL	SS	SS	LL	LL	LL	LL	SS
2	rs3676033	SS	LL	LL	SS	LL	LL	LL	LL	SS
2	rs13476846	SS	LL	LL	LL	LL	LL	LL	LL	SS
2	rs6209403	SS	LL	LL	LL	LL	LL	LL	LL	SS
2	rs6315439	SS	LL	LL	LL	LL	LL	LL	LL	LL
2	rs13476860	SS	LL	LL	LL	LL	LL	LL	LL	SS
2	gnf02.161.674	SS	LL	LL	SS	LL	LL	LL	LL	SS
2	rs6204920	SS	LL	LL	SS	LL	LL	LL	LL	SS
2	rs3673613	SS	LL	LL	SS	LL	LL	LL	LL	SS
2	rs13476894	SS	LL	LL	LL	LL	LL	LL	LL	SS
2	rs6160839	SS	LL	LL	LL	LL	SS	LL	LL	SS
2	rs13476925	SS	LL	SS	LL	LL	SS	LL	LL	SS
2	rs8238755	SS	LL	SS	LL	LL	SS	LL	LL	SS
2	rs13476936	SS	LL	LL	LL	LL	SS	LL	LL	SS
3	rs13476950	SS	LL	LL	SS	LL	LL	SS	SS	SS
3	rs13477017	SS	LL	LL	SS	SS	LL	SS	SS	SS

3	rs3677132	SS	LL	LL	SS	SS	LL	LL	LL	SS
3	CEL-3_31146697	SS	LL	LL	SS	SS	LL	SS	LL	LL
3	rs6351323	SS	LL	LL	SS	SS	LL	SS	LL	LL
3	rs3720738	SS	LL	LL	SS	LL	LL	SS	LL	LL
3	rs13477066	SS	LL	LL	SS	SS	LL	SS	LL	LL
3	rs6335414	SS	LL	SS	SS	SS	LL	SS	SS	LL
3	rs13477178	SS	LL	SS	SS	SS	LL	SS	SS	LL
3	rs3659688	SS	LL	SS	SS	SS	LL	SS	SS	LL
3	gnf03.073.308	SS	LL	SS	SS	SS	LL	LL	SS	LL
3	rs3659866	SS	LL	SS	SS	LL	LL	LL	SS	LL
3	rs13477230	SS	LL	SS	SS	LL	LL	LL	SS	LL
3	rs6243021	SS	LL	SS	SS	LL	LL	LL	SS	LL
3	rs13477242	SS	LL	SS	SS	LL	LL	LL	SS	LL
3	rs13475064	SS	LL	SS	SS	LL	LL	LL	SS	SS
3	rs13477254	SS	LL	SS	SS	LL	LL	LL	LL	SS
3	rs13459185	SS	LL	SS	SS	LL	LL	LL	LL	SS
3	rs3671622	SS	LL	SS	SS	LL	SS	LL	LL	SS
3	rs8256683	SS	LL	SS	LL	LL	SS	LL	LL	SS
3	gnf03.117.090	SS	LL	LL	LL	LL	SS	LL	LL	SS
3	rs13477355	SS	LL	SS	LL	LL	SS	LL	LL	SS
3	rs13477379	SS	LL	SS	LL	LL	SS	LL	LL	SS
3	rs3707706	SS	LL	SS	LL	LL	LL	LL	LL	SS
3	rs13477391	SS	LL	SS	SS	LL	LL	LL	LL	SS
3	rs3658914	SS	LL	SS	SS	SS	LL	LL	LL	SS
3	rs13477410	SS	LL	SS	SS	SS	LL	LL	LL	SS
3	rs13477421	SS	LL	SS	SS	LL	LL	LL	LL	SS
3	CEL-3_137067761	SS	LL	SS	SS	LL	LL	LL	LL	SS
3	rs13477529	SS	LL	SS	SS	LL	LL	LL	LL	SS
3	rs13477477	SS	LL	SS	SS	LL	LL	LL	LL	SS
3	rs3695386	SS	LL	SS	SS	LL	LL	LL	SS	SS
4	rs13477541	SS	LL	LL	LL	SS	LL	SS	SS	SS
4	rs13477546	SS	LL	SS	LL	SS	LL	SS	SS	SS
4	rs6324271	SS	LL	SS	LL	SS	LL	SS	SS	SS
4	rs13477576	SS	LL	SS	SS	SS	LL	SS	SS	SS
4	rs13477595	SS	LL	SS	SS	LL	LL	SS	SS	SS
4	rs13477623	SS	LL	SS	SS	SS	LL	SS	SS	SS
4	rs3719299	SS	LL	SS	SS	SS	LL	SS	LL	SS
4	rs3684104	SS	LL	LL	SS	SS	LL	SS	LL	SS
4	rs13477678	SS	LL	LL	SS	SS	LL	SS	LL	LL
4	rs3707178	SS	LL	LL	SS	SS	LL	SS	LL	LL
4	rs6269326	SS	LL	LL	SS	SS	LL	SS	LL	LL
4	rs13477711	SS	LL	LL	SS	SS	LL	SS	LL	LL

4	rs13477725	SS	LL	LL	SS	SS	LL	SS	SS	LL
4	rs6370644	SS	LL	SS	SS	SS	LL	SS	SS	LL
4	rs13477769	SS	LL	SS	SS	SS	LL	SS	SS	LL
4	rs6255772	SS	LL	SS	SS	SS	LL	LL	SS	LL
4	mCV22668736	SS	LL	SS	SS	SS	LL	LL	SS	LL
4	rs3022993	SS	LL	SS	SS	SS	LL	LL	SS	LL
4	rs3678308	SS	LL	SS	SS	SS	LL	LL	SS	LL
4	rs13477968	SS	LL	SS	SS	SS	LL	LL	SS	LL
4	rs4224808	SS	LL	SS	SS	SS	LL	LL	SS	LL
4	rs3663950	SS	LL	SS	SS	LL	LL	LL	SS	SS
4	rs3023025	SS	LL	SS	SS	LL	LL	SS	SS	SS
4	rs4136314	SS	LL	SS	SS	LL	LL	SS	SS	SS
4	rs3696703	SS	LL	SS	SS	LL	LL	LL	SS	LL
5	rs13478093	SS	LL	SS	LL	SS	LL	LL	SS	SS
5	rs3709946	SS	LL	SS	LL	SS	LL	LL	SS	SS
5	rs3676096	SS	LL	LL	LL	SS	LL	LL	SS	SS
5	rs3714258	SS	LL	LL	LL	SS	LL	LL	SS	SS
5	rs13478133	SS	LL	LL	LL	SS	LL	LL	LL	SS
5	rs13478148	SS	LL	LL	LL	SS	LL	LL	LL	SS
5	rs13478204	SS	LL	SS	SS	SS	LL	LL	LL	SS
5	rs13478217	SS	LL	SS	SS	SS	LL	LL	LL	SS
5	rs6248036	SS	LL	LL	SS	LL	LL	LL	LL	SS
5	rs3711950	SS	LL	LL	SS	LL	LL	LL	LL	SS
5	rs6267669	SS	LL	SS	SS	LL	LL	LL	LL	SS
5	rs3684754	SS	LL	SS	SS	LL	LL	LL	LL	SS
5	rs6409508	SS	LL	SS	SS	SS	LL	LL	LL	SS
5	gnf05.069.163	SS	LL	SS	SS	SS	LL	LL	LL	SS
5	rs6221589	SS	LL	SS	SS	SS	LL	SS	LL	SS
5	rs3658150	SS	LL	SS	LL	SS	LL	SS	LL	SS
5	rs13478355	SS	LL	SS	LL	SS	LL	SS	SS	SS
5	rs13478392	SS	LL	SS	SS	SS	LL	SS	SS	SS
5	rs3661241	SS	LL	SS	SS	SS	LL	SS	SS	SS
5	rs13478473	SS	LL	SS	SS	SS	LL	SS	SS	SS
5	mCV22554962	SS	LL	SS	SS	SS	LL	SS	SS	SS
5	rs3662655	SS	LL	SS	SS	SS	LL	LL	SS	SS
5	rs13478509	SS	LL	SS	LL	SS	LL	LL	SS	SS
5	rs3701266	SS	LL	SS	LL	SS	LL	SS	SS	SS
5	rs13478522	SS	LL	SS	LL	SS	LL	SS	SS	SS
5	rs6377710	SS	LL	LL	LL	SS	LL	LL	SS	SS
5	rs13478540	SS	LL	LL	LL	LL	LL	SS	SS	SS
5	rs3711751	SS	LL	LL	LL	LL	LL	SS	SS	SS
5	rs3023061	SS	LL	LL	LL	SS	SS	LL	LL	SS

5	mCV25009162	SS	LL	LL	LL	SS	LL	LL	LL	SS
5	rs13478589	SS	LL	LL	LL	SS	LL	SS	LL	SS
5	rs3718776	SS	LL	LL	LL	SS	LL	SS	LL	SS
6	rs13478602	SS	LL	SS	LL	LL	LL	SS	SS	LL
6	rs13478612	SS	LL	SS	LL	LL	LL	SS	SS	LL
6	CEL-6_10519419	SS	LL	SS	LL	LL	LL	SS	SS	SS
6	rs3678711	SS	LL	SS	LL	LL	LL	SS	SS	SS
6	rs13478641	SS	LL	SS	LL	LL	LL	SS	SS	SS
6	gnf06.016.989	SS	LL	LL	LL	LL	LL	SS	SS	SS
6	rs3684494	SS	LL	LL	LL	LL	LL	SS	SS	SS
6	rs13478676	SS	LL	LL	LL	LL	LL	SS	SS	SS
6	rs13478681	SS	LL	LL	LL	LL	LL	SS	LL	LL
6	rs6297560	SS	LL	LL	LL	LL	LL	SS	LL	LL
6	gnf06.032.524	SS	LL	LL	SS	LL	LL	SS	LL	LL
6	rs13478717	SS	LL	LL	SS	LL	LL	SS	LL	LL
6	rs13478730	SS	LL	LL	SS	LL	LL	SS	LL	SS
6	rs3684860	SS	LL	LL	SS	LL	LL	SS	LL	LL
6	rs3023069	SS	LL	SS	SS	SS	LL	SS	LL	SS
6	rs13478762	SS	LL	SS	SS	SS	LL	SS	LL	SS
6	gnf06.058.959	SS	LL	SS	SS	SS	LL	SS	LL	LL
6	rs13478818	SS	LL	SS	SS	SS	LL	SS	LL	LL
6	rs6411497	SS	LL	SS	SS	SS	LL	SS	LL	LL
6	rs13459097	SS	LL	SS	SS	SS	SS	SS	LL	LL
6	rs3677567	SS	LL	SS	SS	SS	SS	SS	LL	SS
6	rs6156752	SS	LL	SS	LL	SS	SS	SS	LL	SS
6	rs6223362	SS	LL	SS	LL	SS	SS	SS	LL	SS
6	gnf06.092.758	SS	LL	SS	LL	SS	SS	SS	LL	SS
6	rs13478949	SS	LL	SS	LL	LL	SS	SS	LL	LL
6	rs6204829	SS	LL	SS	SS	LL	SS	SS	LL	LL
6	rs13478999	SS	LL	SS	SS	LL	SS	SS	LL	LL
6	rs3722157	SS	LL	SS	SS	LL	SS	SS	LL	LL
6	CEL-6_127435117	SS	LL	SS	LL	LL	SS	SS	LL	LL
6	rs13479024	SS	LL	SS	LL	LL	SS	SS	LL	LL
6	rs6387265	SS	LL	LL	LL	LL	SS	SS	LL	LL
7	gnf07.010.101	SS	LL	LL	SS	SS	LL	LL	LL	LL
7	UT_7_14.584506	SS	LL	LL	SS	SS	LL	LL	LL	SS
7	rs13479154	SS	LL	LL	SS	SS	LL	SS	LL	SS
7	rs13479171	SS	LL	LL	SS	SS	LL	SS	LL	SS
7	rs3694031	SS	LL	LL	LL	SS	LL	SS	LL	SS
7	rs6217275	SS	LL	LL	LL	LL	LL	SS	LL	SS
7	gnf07.032.360	SS	LL	LL	LL	LL	LL	SS	LL	SS
7	rs3717293	SS	LL	LL	LL	LL	LL	SS	LL	SS

7	rs3679779	SS	LL	LL	LL	LL	LL	SS	LL	SS
7	rs3667441	SS	LL	SS	LL	LL	LL	SS	LL	SS
7	rs3723790	SS	LL	SS	LL	LL	LL	SS	LL	SS
7	rs3676254	SS	LL	SS	LL	LL	LL	SS	LL	SS
7	rs13479342	SS	LL	SS	LL	LL	LL	LL	LL	SS
7	rs13479347	SS	LL	SS	LL	LL	LL	LL	LL	SS
7	rs8248433	SS	LL	SS	LL	LL	LL	LL	LL	LL
7	rs6213614	SS	LL	SS	LL	LL	LL	LL	LL	LL
7	UT_7_90.803899	SS	LL	SS	LL	LL	LL	LL	LL	LL
7	rs13479470	SS	LL	SS	LL	LL	LL	LL	LL	LL
7	rs3656074	SS	LL	SS	LL	LL	LL	LL	LL	LL
7	gnf07.120.460	SS	LL	SS	LL	LL	LL	LL	SS	LL
7	CEL-7_116160192	SS	LL	SS	LL	LL	LL	LL	SS	SS
7	rs8236684	SS	LL	SS	LL	LL	SS	SS	SS	SS
7	CEL-7_126301023	SS	LL	LL	LL	SS	LL	SS	SS	SS
7	rs6299045	SS	LL	LL	LL	SS	LL	SS	SS	SS
7	rs3702894	SS	LL	LL	LL	SS	LL	SS	LL	SS
7	rs6216320	SS	LL	LL	LL	SS	LL	SS	LL	SS
8	rs6273176	SS	LL	LL	LL	SS	LL	LL	LL	SS
8	rs13479601	SS	LL	LL	LL	SS	LL	LL	LL	SS
8	rs6288205	SS	LL	LL	LL	SS	LL	LL	LL	LL
8	rs13479628	SS	LL	LL	LL	SS	LL	LL	SS	LL
8	CEL-8_25677705	SS	LL	LL	SS	SS	LL	LL	SS	LL
8	rs13479769	SS	LL	LL	SS	SS	SS	SS	SS	LL
8	rs13479784	SS	LL	LL	LL	SS	SS	SS	SS	LL
8	rs3672639	SS	LL	LL	LL	LL	SS	SS	SS	LL
8	rs6394046	SS	LL	LL	LL	LL	SS	SS	SS	LL
8	rs13479873	SS	LL	LL	LL	SS	SS	LL	SS	LL
8	rs13479879	SS	LL	SS	LL	SS	SS	SS	SS	LL
8	rs3705695	SS	LL	LL	SS	SS	SS	SS	SS	LL
8	rs6374927	SS	LL	LL	SS	SS	SS	SS	SS	LL
8	rs3675125	SS	LL	SS	SS	SS	SS	SS	SS	LL
8	rs8249856	SS	LL	SS	SS	SS	SS	SS	SS	LL
9	rs13480065	SS	LL	SS	SS	LL	LL	LL	SS	LL
9	rs13480112	SS	LL	LL	SS	LL	LL	LL	SS	LL
9	rs6182405	SS	LL	LL	SS	LL	LL	LL	SS	LL
9	petM-05537-1	SS	LL	LL	SS	LL	LL	LL	LL	LL
9	CEL-9_29909656	SS	LL	LL	SS	LL	LL	LL	LL	LL
9	rs13480130	SS	LL	LL	LL	SS	LL	LL	LL	LL
9	rs3676086	SS	LL	SS	LL	SS	LL	LL	LL	SS
9	rs13480166	SS	LL	SS	LL	SS	LL	LL	SS	SS
9	gnf09.044.276	SS	LL	SS	LL	SS	LL	SS	SS	SS

9	rs13480191	SS	LL	SS	LL	LL	LL	SS	SS	SS
9	rs8259443	SS	LL	SS	LL	LL	LL	SS	LL	SS
9	rs13480218	SS	LL	SS	LL	LL	LL	SS	LL	SS
9	rs13480227	SS	LL	SS	LL	LL	LL	LL	LL	SS
9	rs13480247	SS	LL	SS	LL	LL	LL	LL	LL	SS
9	rs13480258	SS	LL	SS	LL	LL	LL	LL	LL	LL
9	rs3703045	SS	LL	LL	LL	LL	LL	LL	LL	LL
9	rs3724833	SS	LL	LL	LL	LL	LL	LL	LL	LL
9	rs3670195	SS	LL	LL	LL	SS	LL	LL	LL	LL
9	rs13480318	SS	LL	LL	LL	SS	LL	LL	LL	LL
9	gnf09.087.298	SS	LL	LL	LL	SS	LL	LL	LL	LL
9	rs13480387	SS	LL	LL	LL	SS	LL	LL	LL	LL
9	rs3711089	SS	LL	LL	LL	SS	LL	LL	LL	LL
9	rs6320810	SS	LL	LL	LL	SS	LL	LL	LL	SS
9	rs3669563	SS	LL	LL	LL	SS	SS	LL	LL	LL
9	rs13480454	SS	LL	LL	LL	SS	SS	LL	SS	LL
9	rs6299531	SS	LL	LL	LL	SS	SS	LL	SS	LL
9	rs8241505	SS	LL	LL	LL	SS	SS	LL	LL	LL
10	rs13480510	SS	LL	SS	LL	SS	LL	LL	LL	SS
10	rs13480516	SS	LL	SS	LL	SS	LL	LL	LL	SS
10	rs13480527	SS	LL	SS	LL	LL	LL	LL	LL	SS
10	rs3679120	SS	LL	SS	LL	LL	LL	LL	LL	SS
10	rs13480547	SS	LL	SS	LL	LL	LL	LL	LL	SS
10	rs13480601	SS	LL	LL	SS	LL	LL	LL	LL	SS
10	rs3715820	SS	LL	LL	LL	LL	LL	LL	LL	SS
10	rs6186864	SS	LL	LL	LL	LL	LL	SS	LL	SS
10	rs13480630	SS	LL	LL	LL	LL	LL	SS	LL	SS
10	rs13480638	SS	LL	LL	LL	LL	LL	SS	LL	SS
10	rs13480652	SS	LL	LL	LL	LL	LL	SS	LL	SS
10	rs13480678	SS	LL	LL	LL	LL	LL	SS	LL	SS
10	mCV25373751	SS	LL	LL	LL	LL	LL	SS	LL	SS
10	rs3661495	SS	LL	SS	LL	LL	LL	SS	LL	SS
10	rs3704401	SS	LL	SS	LL	LL	LL	SS	LL	SS
10	rs13480739	SS	LL	LL	LL	LL	LL	SS	LL	SS
10	rs13480752	SS	LL	LL	LL	LL	LL	SS	LL	SS
10	rs4228452	SS	LL	LL	LL	LL	LL	SS	LL	SS
10	rs13480773	SS	LL	SS	LL	LL	LL	SS	LL	SS
10	CZECH-10_116791624	SS	LL	SS	LL	LL	SS	SS	LL	SS
10	rs6335076	SS	LL	SS	LL	LL	LL	SS	LL	SS
10	rs6317716	SS	LL	SS	LL	LL	LL	SS	LL	SS
10	rs13480808	SS	LL	LL	LL	LL	LL	SS	LL	SS
11	rs13480835	SS	LL	LL	LL	LL	LL	SS	LL	SS

11	rs6393401	SS	LL	LL	LL	LL	LL	SS	LL	SS
11	rs13480853	SS	LL	LL	LL	LL	LL	LL	LL	SS
11	rs3689494	SS	LL	LL	LL	LL	LL	LL	LL	SS
11	rs3658216	SS	LL	LL	LL	LL	LL	LL	LL	SS
11	rs13480913	SS	LL	LL	LL	LL	LL	LL	LL	SS
11	rs13480918	SS	LL	LL	LL	SS	SS	LL	LL	SS
11	UT_11_29.443258	SS	LL	LL	LL	SS	SS	LL	LL	SS
11	rs3690160	SS	LL	LL	LL	SS	SS	LL	LL	SS
11	rs3660692	SS	LL	LL	LL	SS	SS	LL	LL	SS
11	rs6326787	SS	LL	SS	LL	SS	SS	LL	LL	SS
11	rs6199956	SS	LL	SS	LL	SS	LL	LL	LL	SS
11	rs13481031	SS	LL	SS	LL	SS	LL	LL	LL	SS
11	rs3684076	SS	LL	SS	LL	SS	LL	LL	LL	SS
11	rs13481050	SS	LL	SS	NA	SS	LL	LL	LL	SS
11	rs3714311	SS	LL	LL	NA	SS	LL	SS	LL	SS
11	rs13481075	SS	LL	LL	NA	LL	LL	SS	LL	SS
11	rs13481093	SS	LL	LL	NA	LL	LL	SS	LL	SS
11	CEL-11_73459270	SS	LL	LL	NA	LL	LL	SS	LL	SS
11	rs6178421	SS	LL	LL	NA	LL	LL	SS	LL	SS
11	rs13481123	SS	LL	LL	NA	LL	LL	SS	LL	SS
11	rs3661657	SS	LL	LL	NA	LL	LL	SS	LL	SS
11	rs3688955	SS	LL	LL	NA	LL	LL	SS	SS	LL
11	rs13481161	SS	LL	LL	SS	SS	LL	SS	LL	LL
11	rs3686162	SS	LL	LL	SS	SS	LL	SS	LL	LL
11	rs13474433	SS	LL	LL	SS	SS	LL	SS	LL	LL
11	rs3695865	SS	LL	LL	SS	SS	LL	SS	LL	LL
11	rs6180460	SS	LL	LL	LL	SS	LL	SS	LL	LL
11	rs6407687	SS	LL	LL	LL	SS	LL	SS	SS	LL
11	rs13481210	SS	LL	LL	SS	SS	LL	SS	SS	LL
11	rs13481220	SS	LL	LL	SS	SS	LL	SS	SS	LL
11	rs13481228	SS	LL	LL	SS	SS	LL	SS	SS	SS
11	rs13481233	SS	LL	LL	SS	SS	LL	SS	SS	SS
11	gnf11.121.400	SS	LL	LL	SS	SS	LL	SS	SS	SS
11	rs3662930	SS	LL	LL	SS	SS	LL	LL	SS	SS
11	gnf11.125.992	SS	LL	LL	LL	SS	LL	LL	LL	SS
11	CEL-11_118234030	SS	LL	LL	SS	SS	LL	LL	LL	SS
12	rs3699421	SS	LL	LL	LL	LL	LL	LL	SS	LL
12	rs13481308	SS	LL	LL	LL	LL	LL	SS	SS	LL
12	rs6328018	SS	LL	LL	LL	LL	LL	LL	SS	LL
12	UT_12_24.561109	SS	LL	LL	LL	LL	LL	LL	SS	SS
12	rs3695382	SS	LL	LL	LL	LL	LL	LL	LL	SS
12	rs13481408	SS	LL	LL	SS	LL	LL	LL	LL	SS

12	rs6317361	SS	LL	LL	SS	LL	LL	LL	LL	LL
12	gnf12.053.286	SS	LL	LL	SS	LL	LL	LL	LL	LL
12	rs3660822	SS	LL	LL	SS	LL	LL	SS	LL	LL
12	rs6335879	SS	LL	SS	SS	LL	LL	SS	LL	LL
12	rs3687032	SS	LL	SS	SS	LL	LL	SS	LL	LL
12	rs3709008	SS	LL	SS	SS	LL	LL	SS	LL	LL
12	rs3682382	SS	LL	SS	SS	LL	LL	SS	LL	LL
12	rs3654718	SS	LL	SS	SS	LL	LL	SS	LL	LL
12	rs13481561	SS	LL	SS	SS	LL	LL	SS	LL	LL
12	rs6263380	SS	LL	SS	SS	LL	LL	SS	LL	LL
12	rs13481571	SS	LL	SS	LL	LL	LL	SS	LL	LL
12	rs3711162	SS	LL	SS	LL	LL	LL	SS	LL	LL
12	rs6288403	SS	LL	SS	LL	LL	LL	SS	LL	LL
12	rs13481592	SS	LL	LL	SS	LL	LL	SS	LL	SS
12	rs3679514	SS	LL	LL	SS	LL	LL	SS	LL	SS
12	rs13481618	SS	LL	LL	SS	SS	LL	SS	LL	SS
12	rs13481632	SS	LL	LL	SS	SS	LL	SS	LL	SS
12	rs13481641	SS	LL	LL	SS	SS	LL	SS	SS	SS
12	rs13481651	SS	LL	LL	SS	LL	LL	SS	SS	SS
12	rs4229612	SS	LL	LL	LL	LL	LL	SS	SS	SS
13	rs6215262	SS	LL	LL	NA	LL	SS	SS	SS	SS
13	rs13481702	SS	LL	SS	NA	LL	SS	SS	SS	SS
13	rs13481706	SS	LL	SS	NA	LL	SS	SS	SS	SS
13	rs3678616	SS	LL	LL	NA	LL	SS	SS	SS	SS
13	gnf13.016.175	SS	LL	SS	NA	LL	SS	SS	SS	SS
13	rs4229685	SS	LL	SS	NA	LL	LL	SS	SS	LL
13	rs3724709	SS	LL	SS	LL	SS	LL	SS	SS	LL
13	gnf13.038.133	SS	LL	SS	LL	SS	LL	SS	SS	LL
13	rs6271232	SS	LL	SS	LL	LL	LL	SS	SS	SS
13	rs3688207	SS	LL	LL	LL	LL	LL	SS	SS	SS
13	rs13481823	SS	LL	LL	LL	LL	LL	SS	SS	LL
13	gnf13.057.762	SS	LL	LL	LL	LL	LL	SS	SS	LL
13	rs6410679	SS	LL	LL	LL	LL	LL	SS	SS	LL
13	rs6381045	SS	LL	LL	LL	LL	LL	SS	SS	SS
13	rs3721965	SS	LL	LL	LL	LL	LL	SS	SS	LL
13	rs13481886	SS	LL	LL	SS	LL	LL	SS	SS	LL
13	rs13481892	SS	LL	LL	SS	LL	LL	SS	SS	LL
13	mCV24625340	SS	LL	LL	SS	LL	LL	SS	SS	LL
13	gnf13.092.499	SS	LL	LL	SS	LL	SS	SS	SS	LL
13	rs13481958	SS	LL	LL	SS	LL	SS	SS	SS	SS
13	rs3688959	SS	LL	LL	NA	LL	SS	SS	LL	SS
13	rs6389588	SS	LL	LL	NA	LL	LL	SS	LL	SS

13	rs13482000	SS	LL	SS	LL	LL	LL	SS	SS	SS
13	mCV24886326	SS	LL	LL	LL	SS	LL	SS	SS	SS
13	rs6247696	SS	LL	LL	LL	LL	LL	SS	LL	SS
13	rs6397687	SS	LL	LL	LL	LL	LL	SS	LL	SS
14	rs3687889	SS	LL	LL	LL	SS	LL	LL	SS	LL
14	rs6397486	SS	LL	LL	LL	LL	LL	LL	SS	LL
14	mCV23128760	SS	LL	LL	LL	LL	LL	LL	SS	LL
14	rs3701623	SS	LL	SS	LL	LL	LL	SS	LL	LL
14	rs13482262	SS	LL	SS	LL	LL	LL	SS	LL	LL
14	rs6352085	SS	LL	SS	LL	LL	LL	SS	LL	SS
14	rs13482276	SS	LL	SS	LL	LL	LL	SS	LL	SS
14	rs13482281	SS	LL	SS	LL	LL	LL	SS	SS	SS
14	rs13482296	SS	LL	SS	LL	LL	LL	SS	SS	SS
14	rs6211694	SS	LL	SS	LL	SS	LL	SS	SS	LL
14	CEL-14_110783830	SS	LL	SS	LL	SS	LL	SS	SS	SS
14	rs3707842	SS	LL	SS	LL	SS	LL	SS	SS	LL
14	gnf14.117.278	SS	LL	SS	LL	SS	LL	LL	SS	LL
14	rs13482416	SS	LL	SS	LL	LL	LL	LL	SS	LL
15	rs13459176	SS	LL	LL	SS	LL	LL	LL	SS	LL
15	rs13482431	SS	LL	LL	LL	LL	LL	LL	SS	LL
15	rs13482446	SS	LL	LL	LL	LL	SS	LL	SS	LL
15	CEL-15_15919629	SS	LL	LL	LL	LL	SS	LL	SS	LL
15	rs13482461	SS	LL	LL	LL	LL	SS	LL	SS	LL
15	rs13482486	SS	LL	SS	LL	LL	SS	LL	SS	LL
15	rs3670581	SS	LL	SS	LL	LL	SS	LL	SS	LL
15	rs13482498	SS	LL	SS	LL	SS	SS	LL	SS	LL
15	rs3088525	SS	LL	SS	LL	SS	SS	SS	SS	LL
15	rs6188239	SS	LL	SS	LL	SS	SS	SS	SS	LL
15	rs3695416	SS	LL	SS	LL	SS	SS	SS	SS	LL
15	rs3677296	SS	LL	SS	LL	SS	SS	LL	SS	LL
15	rs13482536	SS	LL	SS	LL	SS	SS	LL	SS	LL
15	rs13482541	SS	LL	SS	LL	SS	SS	LL	SS	LL
15	rs3660290	SS	LL	LL	LL	SS	SS	LL	SS	SS
15	rs13482589	SS	LL	LL	LL	SS	SS	LL	SS	SS
15	rs13482595	SS	LL	SS	LL	SS	SS	LL	SS	SS
15	rs13482612	SS	LL	SS	LL	SS	SS	LL	LL	SS
15	rs13482618	SS	LL	LL	LL	SS	SS	LL	LL	SS
15	rs6197332	SS	LL	SS	LL	SS	SS	LL	LL	SS
15	rs13482642	SS	LL	SS	LL	SS	SS	LL	LL	SS
15	rs6287697	SS	LL	SS	LL	SS	SS	LL	LL	SS
15	rs13482711	SS	LL	SS	LL	SS	SS	LL	LL	SS
15	rs13482719	SS	LL	SS	LL	SS	LL	LL	LL	SS

15	rs13482726	SS	LL	SS	LL	SS	LL	LL	LL	LL
15	rs13482732	SS	LL	SS	LL	SS	SS	LL	LL	LL
15	rs3708604	SS	LL	SS	LL	SS	SS	LL	LL	LL
15	rs13482741	SS	LL	SS	LL	SS	SS	LL	LL	LL
16	rs4152386	SS	LL	SS	SS	LL	LL	LL	SS	SS
16	rs4165119	SS	LL	LL	SS	LL	LL	LL	LL	SS
16	rs6294027	SS	LL	SS	SS	LL	LL	LL	LL	SS
16	rs4172338	SS	LL	SS	SS	LL	LL	LL	LL	SS
16	rs4173709	SS	LL	SS	SS	LL	SS	LL	LL	SS
16	rs4175608	SS	LL	LL	SS	LL	SS	LL	LL	SS
16	rs4177203	SS	LL	LL	SS	SS	SS	LL	LL	SS
16	rs4179117	SS	LL	LL	SS	SS	SS	LL	SS	SS
16	rs4184315	SS	LL	LL	LL	SS	SS	LL	SS	SS
16	rs4185176	SS	LL	LL	LL	SS	SS	SS	SS	SS
16	rs6271301	SS	LL	LL	SS	SS	SS	SS	SS	SS
16	rs4192132	SS	LL	LL	SS	SS	SS	SS	SS	SS
16	rs4197416	SS	LL	LL	SS	SS	SS	LL	SS	SS
16	rs4197725	SS	LL	LL	SS	SS	SS	SS	SS	SS
16	rs4202372	SS	LL	LL	SS	SS	SS	LL	SS	SS
16	rs4203891	SS	LL	LL	SS	SS	SS	LL	SS	SS
16	rs4205524	SS	LL	LL	SS	SS	SS	SS	SS	SS
16	rs4211515	SS	LL	NA	SS	LL	SS	SS	SS	SS
16	rs4211731	SS	LL	NA	SS	LL	SS	SS	SS	SS
16	rs4219239	SS	LL	SS	SS	LL	LL	LL	SS	SS
16	rs4219897	SS	LL	SS	SS	LL	LL	LL	SS	SS
17	rs3724616	SS	LL	SS	LL	SS	LL	LL	SS	SS
17	rs3702484	SS	LL	SS	LL	SS	LL	LL	SS	LL
17	rs4231344	SS	LL	LL	SS	SS	LL	SS	SS	LL
17	rs3090500	SS	LL	LL	SS	SS	LL	SS	SS	LL
17	rs4136360	SS	LL	LL	SS	SS	LL	SS	SS	LL
17	rs6358703	SS	LL	LL	SS	SS	LL	SS	SS	LL
17	rs13482947	SS	LL	LL	LL	SS	LL	SS	SS	SS
17	gnf17.035.152	SS	LL	LL	LL	SS	LL	LL	SS	SS
17	rs13482968	SS	LL	LL	LL	SS	LL	LL	SS	SS
17	rs13482973	SS	LL	SS	LL	SS	LL	LL	SS	SS
17	rs3090988	SS	LL	SS	LL	SS	LL	LL	SS	SS
17	mCV22888090	SS	LL	LL	LL	LL	LL	SS	SS	SS
17	rs13483075	SS	LL	LL	LL	LL	LL	SS	SS	SS
17	rs6322076	SS	LL	LL	LL	LL	SS	SS	SS	SS
17	rs6386440	SS	LL	LL	LL	LL	SS	SS	SS	SS
17	rs3684732	SS	LL	LL	SS	LL	SS	SS	SS	SS
17	rs3701338	SS	LL	LL	SS	LL	SS	SS	SS	SS

17	rs13483144	SS	LL	LL	SS	LL	SS	LL	SS	SS
18	rs13483183	SS	LL	LL	LL	SS	SS	SS	LL	LL
18	rs13483200	SS	LL	LL	LL	SS	SS	SS	LL	LL
18	rs13483210	SS	LL	LL	LL	SS	SS	SS	LL	SS
18	mCV23617245	SS	LL	LL	LL	SS	SS	SS	LL	SS
18	rs6194744	SS	LL	LL	LL	SS	LL	SS	LL	SS
18	rs13483262	SS	LL	LL	LL	SS	LL	SS	LL	SS
18	rs13483277	SS	LL	SS	LL	SS	LL	SS	LL	SS
18	CEL-18_32158369	SS	LL	SS	LL	SS	LL	SS	SS	SS
18	rs3675531	SS	LL	SS	LL	SS	SS	SS	SS	LL
18	gnf18.033.953	SS	LL	SS	SS	SS	SS	SS	SS	SS
18	rs13483319	SS	LL	SS	SS	SS	SS	SS	SS	SS
18	rs6313313	SS	LL	SS	SS	SS	SS	SS	SS	SS
18	rs3676196	SS	LL	SS	SS	SS	SS	SS	SS	SS
18	rs13483340	SS	LL	SS	SS	LL	SS	SS	SS	SS
18	rs6184541	SS	LL	SS	SS	LL	LL	SS	SS	SS
18	rs3654438	SS	LL	LL	SS	SS	LL	SS	SS	SS
18	rs3688789	SS	LL	LL	SS	SS	LL	SS	SS	SS
18	rs3716803	SS	LL	LL	SS	SS	LL	SS	SS	SS
18	rs13483423	SS	LL	LL	SS	SS	LL	SS	SS	SS
18	gnf18.069.928	SS	LL	LL	SS	SS	LL	SS	SS	SS
18	rs6302629	SS	LL	LL	LL	SS	LL	SS	SS	SS
18	rs3705890	SS	LL	SS	LL	SS	LL	SS	SS	SS
18	rs3671707	SS	LL	SS	LL	SS	LL	SS	SS	SS
18	rs13483466	SS	LL	SS	LL	SS	LL	SS	SS	SS
19	rs3713033	SS	LL	SS	SS	LL	SS	SS	LL	LL
19	rs13483526	SS	LL	SS	LL	LL	SS	SS	LL	LL
19	rs6316813	SS	LL	SS	LL	LL	SS	SS	LL	LL
19	rs6307076	SS	LL	SS	LL	LL	SS	LL	LL	LL
19	rs6342493	SS	LL	LL	SS	LL	SS	SS	LL	SS
19	rs6291559	SS	LL	LL	SS	LL	SS	SS	LL	SS
19	rs3714482	SS	LL	LL	SS	LL	LL	SS	LL	SS
19	CEL-19_32349880	SS	LL	LL	SS	LL	SS	SS	LL	SS
19	rs13483650	SS	LL	LL	LL	SS	SS	SS	LL	SS
19	CEL-19_48242857	SS	LL	LL	LL	SS	SS	SS	LL	SS
19	rs13483677	SS	LL	SS	LL	SS	SS	SS	LL	SS
19	rs3711945	SS	LL	SS	SS	SS	SS	SS	LL	SS
X	rs13483712	SS	LL	SS	SS	LL	LL	LL	LL	SS
X	rs13483724	SS	LL	LL	LL	LL	LL	LL	LL	SS
X	rs13483777	SS	LL	LL	LL	LL	LL	LL	LL	LL
X	rs13483884	SS	LL	LL	LL	LL	LL	SS	LL	LL
X	rs13483899	SS	LL	SS	LL	LL	LL	SS	LL	LL

X	gnfX.084.751	SS	LL	LL	LL	LL	LL	SS	LL	LL
X	gnfX.086.039	SS	LL	LL	LL	LL	LL	SS	SS	LL
X	rs13483951	SS	LL	LL	LL	LL	LL	SS	SS	LL
X	rs6205221	SS	LL	LL	LL	LL	LL	SS	SS	LL
X	rs13484003	SS	LL	LL	LL	LL	LL	SS	SS	LL
X	gnfX.118.600	SS	LL	LL	LL	LL	LL	SS	LL	LL
X	rs13484038	SS	LL	LL	LL	LL	LL	SS	LL	LL
X	gnfX.124.190	SS	LL	LL	LL	LL	LL	SS	LL	LL
X	CEL-X_133525088	SS	LL	LL	LL	LL	LL	SS	LL	LL
X	rs13484050	SS	LL	LL	LL	LL	LL	LL	LL	LL
X	rs13484093	SS	LL	LL	LL	LL	LL	LL	LL	LL
X	rs13484094	SS	LL	LL	LL	LL	LL	LL	LL	LL

CHR	SNP	22	23	31	33	35	38	39	45	46	48
1	rs3683945	LL	SS	LL	LL	SS	LL	LL	SS	LL	LL
1	rs13475748	LL	SS	LL	LL	SS	LL	LL	SS	LL	LL
1	rs13475774	LL	SS	LL	LL	LL	LL	LL	SS	LL	LL
1	rs13475816	LL	SS	LL	LL	LL	SS	SS	SS	LL	LL
1	rs3685569	LL	SS	LL	LL	LL	SS	SS	SS	LL	SS
1	gnf01.037.906	LL	SS	LL	LL	LL	SS	SS	SS	LL	LL
1	rs3663706	LL	SS	LL	LL	SS	SS	SS	SS	LL	LL
1	rs13475837	LL	SS	LL	LL	LL	SS	SS	SS	LL	LL
1	rs13475863	LL	SS	LL	SS	LL	SS	SS	SS	LL	SS
1	rs13475874	LL	SS	LL	SS	LL	SS	SS	SS	SS	SS
1	petAF067836-350A-1	LL	LL	LL	SS	LL	SS	SS	SS	SS	SS
1	rs13475894	LL	LL	LL	SS	LL	SS	LL	SS	LL	LL
1	rs13475902	LL	LL	LL	SS	LL	SS	LL	SS	LL	LL
1	rs13475903	LL	LL	LL	LL	LL	SS	LL	SS	LL	LL
1	rs13475931	LL	LL	LL	SS	LL	SS	LL	SS	LL	LL
1	rs3667200	LL	LL	LL	LL	LL	SS	LL	SS	LL	LL
1	rs13475970	LL	LL	LL	LL	LL	SS	LL	SS	LL	LL
1	rs3664800	LL	LL	LL	NA	LL	SS	LL	SS	LL	LL
1	rs6342650	LL	LL	LL	NA	LL	SS	LL	SS	LL	LL
1	rs13476036	LL	LL	LL	NA	LL	SS	LL	SS	LL	SS
1	mCV22824651	LL	LL	LL	NA	LL	SS	LL	SS	LL	SS
1	rs3719973	LL	LL	LL	NA	LL	SS	LL	SS	LL	LL
1	rs3686671	LL	LL	LL	NA	LL	SS	LL	SS	LL	LL
1	rs3691374	LL	LL	LL	SS	LL	SS	LL	SS	SS	LL
1	rs13476122	LL	LL	LL	SS	LL	SS	LL	SS	LL	LL
1	rs13476125	LL	LL	LL	SS	SS	SS	LL	SS	LL	SS
1	rs6250257	LL	SS	LL	SS	SS	SS	LL	SS	LL	SS
1	rs13476136	LL	SS	LL	SS	LL	SS	LL	SS	LL	SS
1	rs6186115	LL	SS	LL	SS	LL	SS	LL	SS	LL	LL
1	gnf01.157.188	LL	SS	LL	SS	LL	SS	LL	SS	LL	LL
1	rs13476211	LL	LL	LL	SS	LL	SS	LL	SS	LL	SS
1	rs8256589	LL	LL	LL	SS	LL	SS	LL	SS	SS	SS
1	rs8242852	LL	LL	LL	LL	SS	LL	LL	SS	SS	SS
1	rs13476248	LL	LL	LL	LL	SS	LL	LL	SS	SS	LL
1	rs3723788	LL	LL	LL	LL	LL	LL	LL	SS	SS	LL
1	mCV23509126	LL	LL	LL	LL	SS	LL	LL	SS	SS	LL
1	rs6157620	LL	SS	LL	LL	SS	LL	LL	SS	SS	LL
1	rs13476286	LL	SS	LL	LL	SS	LL	LL	SS	SS	LL
1	rs13476293	LL	SS	LL	LL	SS	LL	LL	SS	SS	SS

1	rs13476308	LL	SS	LL	LL	SS	LL	SS	SS	SS	SS
2	rs13476319	SS	LL	LL	SS	LL	SS	SS	LL	LL	LL
2	rs6359983	SS	LL	LL	SS	LL	SS	LL	LL	LL	LL
2	gnf02.007.482	SS	LL	LL	SS	LL	SS	LL	LL	LL	LL
2	rs3688854	SS	LL	LL	LL	SS	LL	SS	LL	LL	LL
2	mCV23209429	SS	LL	LL	LL	SS	LL	SS	SS	LL	SS
2	rs13476425	SS	LL	LL	SS	SS	LL	SS	SS	LL	SS
2	rs6313371	SS	LL	LL	SS	SS	LL	LL	SS	LL	SS
2	rs3680197	SS	LL	LL	SS	SS	LL	LL	SS	LL	SS
2	rs3718711	LL	LL	LL	LL	SS	LL	LL	SS	LL	SS
2	rs3022886	LL	LL	LL	LL	SS	LL	LL	SS	LL	SS
2	rs8263587	LL	LL	SS	SS	SS	LL	SS	SS	LL	SS
2	rs6295014	LL	LL	LL	NA	SS	LL	LL	LL	LL	SS
2	rs3710094	LL	LL	LL	NA	SS	LL	LL	LL	LL	SS
2	CEL-2_79237503	LL	LL	LL	NA	LL	LL	LL	LL	LL	SS
2	rs13476598	SS	LL	LL	NA	LL	LL	LL	LL	LL	SS
2	rs13476637	SS	LL	LL	NA	LL	LL	LL	LL	LL	LL
2	rs13476647	SS	LL	LL	NA	LL	LL	LL	LL	LL	SS
2	rs6406705	SS	LL	LL	NA	LL	LL	LL	LL	LL	SS
2	rs6208879	SS	LL	LL	NA	LL	LL	LL	LL	LL	SS
2	rs13476723	LL	LL	LL	NA	LL	LL	LL	LL	LL	SS
2	rs3725853	LL	LL	LL	NA	SS	LL	LL	LL	LL	SS
2	rs13476761	LL	LL	LL	SS	SS	SS	LL	LL	LL	SS
2	rs13476787	LL	LL	LL	SS	SS	SS	SS	LL	LL	SS
2	rs6249968	LL	LL	LL	SS	SS	SS	LL	LL	LL	SS
2	mCV23169908	LL	LL	LL	SS	SS	SS	LL	LL	LL	SS
2	rs3676033	LL	LL	LL	SS	SS	SS	SS	LL	LL	SS
2	rs13476846	LL	LL	LL	SS	LL	SS	SS	LL	LL	SS
2	rs6209403	LL	LL	LL	SS	LL	SS	LL	LL	LL	SS
2	rs6315439	LL	LL	LL	LL	LL	LL	SS	LL	LL	LL
2	rs13476860	LL	LL	LL	SS	LL	SS	SS	LL	LL	SS
2	gnf02.161.674	LL	LL	LL	SS	SS	SS	SS	LL	LL	SS
2	rs6204920	LL	LL	LL	SS	SS	SS	SS	SS	LL	SS
2	rs3673613	LL	LL	LL	SS	SS	SS	SS	SS	LL	LL
2	rs13476894	LL	LL	LL	SS	LL	SS	SS	SS	LL	LL
2	rs6160839	LL	LL	SS	SS	LL	SS	SS	SS	SS	LL
2	rs13476925	SS	LL	SS	SS	LL	SS	SS	SS	SS	LL
2	rs8238755	SS	LL	SS	LL	LL	LL	SS	SS	SS	LL
2	rs13476936	SS	LL	SS	LL	LL	LL	SS	SS	SS	LL
3	rs13476950	LL	SS	LL	SS	SS	SS	SS	SS	SS	LL
3	rs13477017	LL	SS	LL	SS	SS	SS	SS	SS	SS	LL
3	rs3677132	LL	SS	LL	SS	SS	SS	SS	SS	SS	LL

3	CEL-3_31146697	LL	SS	LL	SS	SS	SS	SS	SS	SS	SS
3	rs6351323	LL	SS	LL	LL	SS	SS	SS	SS	SS	SS
3	rs3720738	LL	SS	LL	LL	SS	SS	SS	SS	LL	SS
3	rs13477066	LL	SS	LL	LL	SS	SS	SS	LL	LL	SS
3	rs6335414	LL	SS	LL	LL	SS	SS	SS	LL	LL	LL
3	rs13477178	LL	SS	LL	LL	SS	SS	SS	LL	LL	LL
3	rs3659688	LL	SS	LL	LL	SS	SS	LL	LL	LL	LL
3	gnf03.073.308	LL	SS	LL	LL	SS	SS	LL	LL	LL	LL
3	rs3659866	LL	SS	LL	LL	SS	SS	LL	LL	LL	LL
3	rs13477230	LL	SS	LL	LL	SS	SS	LL	LL	SS	LL
3	rs6243021	LL	SS	LL	SS	SS	SS	LL	LL	SS	LL
3	rs13477242	SS	SS	LL	SS	SS	SS	LL	LL	SS	LL
3	rs13475064	SS	LL	LL	SS	SS	SS	LL	LL	SS	LL
3	rs13477254	SS	LL	LL	SS	SS	SS	LL	LL	SS	LL
3	rs13459185	LL	LL	LL	SS	SS	SS	LL	LL	SS	SS
3	rs3671622	LL	LL	SS	SS	SS	SS	LL	LL	SS	SS
3	rs8256683	LL	LL	SS	SS	SS	SS	LL	LL	SS	SS
3	gnf03.117.090	LL	LL	SS	SS	SS	SS	LL	LL	SS	SS
3	rs13477355	LL	LL	SS	LL	SS	LL	LL	LL	SS	SS
3	rs13477379	LL	LL	SS	LL	SS	LL	LL	LL	LL	LL
3	rs3707706	LL	SS	LL	LL	SS	LL	LL	LL	LL	LL
3	rs13477391	LL	SS	LL	LL	SS	LL	LL	LL	LL	LL
3	rs3658914	LL	SS	LL	LL	SS	LL	LL	LL	LL	LL
3	rs13477410	LL	SS	LL	SS	SS	SS	LL	LL	LL	LL
3	rs13477421	LL	SS	LL	SS	SS	SS	LL	LL	LL	SS
3	CEL-3_137067761	SS	SS	LL	SS	SS	SS	LL	LL	LL	SS
3	rs13477529	SS	SS	LL	SS	SS	LL	LL	LL	LL	SS
3	rs13477477	SS	SS	LL	SS	SS	LL	LL	LL	SS	SS
3	rs3695386	SS	SS	LL	SS	SS	LL	LL	LL	SS	SS
4	rs13477541	SS	SS	LL	SS	LL	SS	LL	LL	LL	LL
4	rs13477546	SS	SS	LL	SS	LL	SS	LL	LL	LL	LL
4	rs6324271	SS	SS	LL	SS	LL	SS	LL	SS	LL	LL
4	rs13477576	SS	SS	LL	SS	SS	SS	LL	SS	LL	LL
4	rs13477595	SS	SS	LL	SS	SS	SS	LL	SS	LL	SS
4	rs13477623	SS	SS	LL	SS	SS	SS	LL	SS	LL	SS
4	rs3719299	SS	SS	LL	SS	SS	SS	LL	SS	LL	SS
4	rs3684104	SS	SS	LL	SS	SS	SS	LL	SS	LL	SS
4	rs13477678	SS	SS	LL	SS	SS	SS	SS	SS	LL	SS
4	rs3707178	SS	SS	LL	LL	SS	LL	SS	SS	LL	SS
4	rs6269326	SS	SS	LL	LL	SS	LL	SS	SS	LL	LL
4	rs13477711	SS	SS	LL	LL	SS	LL	SS	SS	LL	SS
4	rs13477725	SS	SS	LL	LL	SS	LL	SS	SS	LL	SS

4	rs6370644	SS	SS	LL	LL	SS	LL	SS	SS	LL	SS
4	rs13477769	SS	SS	LL	SS	SS	SS	SS	SS	LL	SS
4	rs6255772	SS	SS	LL	SS	SS	SS	SS	SS	LL	SS
4	mCV22668736	LL	SS	LL	SS	SS	SS	SS	SS	LL	SS
4	rs3022993	LL	SS	LL	LL	SS	SS	SS	SS	LL	LL
4	rs3678308	LL	SS	LL	SS	SS	SS	SS	SS	SS	LL
4	rs13477968	SS	SS	LL	LL	SS	SS	SS	SS	SS	LL
4	rs4224808	SS	SS	LL	LL	SS	SS	SS	SS	SS	SS
4	rs3663950	SS	LL	LL	LL	SS	SS	SS	SS	SS	SS
4	rs3023025	SS	SS	LL	SS	SS	SS	LL	LL	SS	SS
4	rs4136314	SS	LL	LL	SS	SS	SS	LL	LL	SS	SS
4	rs3696703	SS	LL	LL	SS	SS	LL	LL	LL	LL	SS
5	rs13478093	LL	LL	LL	SS	LL	SS	LL	SS	LL	LL
5	rs3709946	LL	LL	LL	SS	LL	SS	LL	SS	SS	LL
5	rs3676096	LL	LL	LL	SS	LL	SS	LL	SS	SS	LL
5	rs3714258	LL	LL	LL	SS	LL	SS	LL	SS	LL	LL
5	rs13478133	LL	LL	LL	SS	LL	SS	LL	SS	LL	SS
5	rs13478148	LL	LL	LL	SS	LL	SS	LL	LL	LL	SS
5	rs13478204	LL	LL	LL	SS	SS	SS	LL	LL	LL	SS
5	rs13478217	SS	LL	LL	SS	SS	SS	LL	LL	SS	SS
5	rs6248036	SS	SS	LL	SS	SS	SS	LL	LL	SS	SS
5	rs3711950	LL	LL	LL	SS	SS	SS	LL	LL	SS	SS
5	rs6267669	LL	LL	LL	SS	SS	SS	LL	LL	LL	SS
5	rs3684754	LL	SS	LL	SS	SS	SS	LL	LL	LL	SS
5	rs6409508	LL	SS	LL	SS	SS	SS	LL	LL	LL	SS
5	gnf05.069.163	LL	LL	LL	SS	SS	SS	LL	LL	LL	SS
5	rs6221589	LL	LL	LL	SS	SS	SS	LL	LL	LL	SS
5	rs3658150	LL	LL	LL	SS	LL	SS	LL	LL	LL	SS
5	rs13478355	LL	LL	LL	SS	LL	SS	LL	LL	LL	SS
5	rs13478392	LL	LL	LL	SS	SS	SS	LL	LL	LL	SS
5	rs3661241	LL	LL	LL	LL	SS	LL	LL	LL	LL	SS
5	rs13478473	SS	LL	LL	LL	SS	LL	LL	LL	LL	SS
5	mCV22554962	SS	LL	LL	SS	SS	SS	LL	LL	LL	SS
5	rs3662655	SS	LL	LL	SS	SS	SS	LL	LL	LL	SS
5	rs13478509	SS	LL	LL	SS	LL	SS	LL	LL	LL	SS
5	rs3701266	SS	LL	LL	SS	LL	SS	LL	LL	LL	SS
5	rs13478522	SS	LL	LL	SS	LL	SS	LL	LL	SS	SS
5	rs6377710	SS	LL	LL	SS	LL	SS	LL	LL	SS	LL
5	rs13478540	SS	LL	LL	LL	LL	LL	LL	LL	SS	LL
5	rs3711751	SS	LL	LL	LL	LL	LL	LL	LL	LL	LL
5	rs3023061	SS	LL	SS	LL	LL	LL	LL	LL	LL	LL
5	mCV25009162	SS	LL	LL	LL	SS	LL	LL	LL	LL	LL

5	rs13478589	SS	SS	LL	LL	SS	LL	SS	LL	LL	SS
5	rs3718776	SS	SS	LL	LL	SS	LL	LL	LL	LL	SS
6	rs13478602	SS	LL	LL	SS	LL	SS	SS	SS	LL	SS
6	rs13478612	SS	LL	LL	SS	LL	SS	SS	SS	LL	NA
6	CEL-6_10519419	SS	LL	LL	SS	LL	SS	SS	SS	LL	NA
6	rs3678711	SS	LL	LL	SS	LL	SS	SS	LL	SS	NA
6	rs13478641	SS	LL	LL	SS	LL	SS	SS	LL	SS	LL
6	gnf06.016.989	SS	LL	LL	SS	LL	SS	SS	LL	SS	LL
6	rs3684494	SS	LL	LL	SS	SS	SS	SS	LL	SS	LL
6	rs13478676	SS	LL	LL	LL	SS	LL	SS	LL	SS	LL
6	rs13478681	SS	LL	LL	LL	SS	LL	SS	LL	SS	LL
6	rs6297560	SS	LL	LL	LL	LL	LL	SS	LL	SS	LL
6	gnf06.032.524	SS	LL	LL	LL	SS	LL	SS	LL	SS	LL
6	rs13478717	SS	LL	LL	LL	SS	LL	SS	LL	SS	SS
6	rs13478730	SS	LL	LL	LL	SS	LL	SS	LL	SS	SS
6	rs3684860	SS	LL	LL	LL	SS	LL	SS	LL	LL	SS
6	rs3023069	SS	LL	LL	LL	SS	LL	SS	LL	LL	SS
6	rs13478762	SS	LL	LL	LL	SS	LL	SS	LL	SS	SS
6	gnf06.058.959	SS	LL	LL	LL	SS	LL	SS	LL	SS	SS
6	rs13478818	SS	LL	LL	LL	SS	LL	SS	SS	LL	SS
6	rs6411497	SS	SS	LL	LL	SS	LL	SS	SS	LL	SS
6	rs13459097	SS	SS	SS	LL	SS	LL	SS	SS	LL	SS
6	rs3677567	SS	SS	SS	LL	SS	LL	SS	SS	LL	SS
6	rs6156752	SS	LL	SS	LL	LL	LL	SS	SS	LL	SS
6	rs6223362	SS	LL	SS	LL	LL	SS	SS	SS	LL	SS
6	gnf06.092.758	SS	LL	SS	LL	LL	SS	LL	SS	LL	SS
6	rs13478949	SS	SS	SS	SS	LL	LL	SS	SS	LL	SS
6	rs6204829	SS	SS	SS	SS	SS	LL	SS	SS	LL	SS
6	rs13478999	SS	SS	SS	SS	SS	LL	SS	SS	LL	SS
6	rs3722157	SS	SS	SS	LL	SS	LL	SS	SS	LL	SS
6	CEL-6_127435117	SS	SS	SS	LL	LL	LL	SS	SS	LL	SS
6	rs13479024	SS	SS	SS	LL	LL	LL	SS	SS	SS	SS
6	rs6387265	SS	SS	SS	LL	LL	LL	SS	SS	SS	SS
7	gnf07.010.101	SS	LL	LL	LL	LL	LL	SS	LL	SS	SS
7	UT_7_14.584506	SS	LL	LL	LL	SS	LL	SS	LL	SS	SS
7	rs13479154	SS	LL	LL	LL	SS	LL	SS	LL	SS	SS
7	rs13479171	SS	LL	LL	LL	SS	LL	SS	LL	SS	LL
7	rs3694031	SS	LL	LL	LL	LL	LL	SS	LL	SS	LL
7	rs6217275	SS	LL	LL	LL	LL	LL	SS	LL	SS	LL
7	gnf07.032.360	SS	LL	LL	LL	LL	LL	SS	LL	SS	LL
7	rs3717293	SS	SS	LL	LL	LL	LL	SS	LL	SS	LL
7	rs3679779	SS	SS	LL	LL	LL	LL	SS	LL	SS	LL

7	rs3667441	SS	SS	LL	LL	LL	LL	SS	LL	SS	LL
7	rs3723790	SS	SS	LL	LL	LL	LL	SS	LL	LL	LL
7	rs3676254	SS	SS	LL	LL	LL	LL	SS	SS	LL	LL
7	rs13479342	SS	SS	LL	LL	LL	LL	SS	SS	LL	LL
7	rs13479347	SS	SS	LL	LL	LL	LL	LL	SS	LL	LL
7	rs8248433	SS	SS	LL	LL	LL	LL	LL	SS	LL	LL
7	rs6213614	SS	SS	LL	LL	LL	LL	SS	SS	LL	LL
7	UT_7_90.803899	LL	SS	LL	LL	LL	LL	SS	SS	LL	LL
7	rs13479470	LL	SS	LL	LL	LL	LL	SS	SS	LL	LL
7	rs3656074	LL	SS	LL	SS	LL	SS	SS	SS	LL	LL
7	gnf07.120.460	LL	SS	LL	SS	LL	SS	LL	SS	LL	LL
7	CEL-7_116160192	LL	SS	LL	SS	LL	SS	LL	SS	LL	LL
7	rs8236684	SS	SS	SS	SS	LL	SS	SS	SS	LL	LL
7	CEL-7_126301023	SS	LL	LL	SS	LL	SS	SS	SS	SS	LL
7	rs6299045	SS	LL	LL	SS	LL	SS	LL	SS	SS	LL
7	rs3702894	SS	LL	LL	SS	LL	SS	LL	SS	SS	LL
7	rs6216320	SS	SS	LL	SS	LL	SS	LL	SS	SS	LL
8	rs6273176	LL	LL	LL	LL	SS	LL	SS	LL	LL	LL
8	rs13479601	LL	LL	LL	LL	LL	LL	SS	LL	LL	LL
8	rs6288205	LL	LL	LL	LL	LL	LL	SS	LL	LL	LL
8	rs13479628	LL	LL	LL	LL	LL	LL	SS	LL	LL	LL
8	CEL-8_25677705	SS	LL	LL	LL	SS	LL	SS	LL	LL	LL
8	rs13479769	LL	LL	SS	LL	SS	LL	SS	LL	LL	LL
8	rs13479784	LL	LL	SS	LL	LL	LL	SS	LL	LL	LL
8	rs3672639	LL	SS	SS	LL	LL	LL	SS	LL	LL	LL
8	rs6394046	LL	LL	SS	LL	LL	LL	SS	LL	LL	LL
8	rs13479873	LL	LL	SS	LL	LL	LL	SS	LL	LL	SS
8	rs13479879	LL	LL	SS	LL	LL	LL	SS	LL	LL	SS
8	rs3705695	LL	LL	SS	SS	SS	SS	SS	LL	SS	SS
8	rs6374927	SS	LL	SS	LL	SS	SS	SS	LL	SS	SS
8	rs3675125	LL	LL	SS	SS	SS	SS	SS	LL	LL	LL
8	rs8249856	LL	LL	SS	SS	SS	LL	SS	LL	LL	LL
9	rs13480065	LL	SS	LL	LL	SS	LL	SS	SS	LL	SS
9	rs13480112	SS	SS	LL	LL	SS	LL	LL	LL	LL	LL
9	rs6182405	SS	SS	LL	LL	SS	LL	LL	LL	LL	SS
9	petM-05537-1	SS	SS	LL	LL	SS	NA	LL	LL	LL	SS
9	CEL-9_29909656	SS	SS	LL	LL	SS	NA	LL	LL	SS	SS
9	rs13480130	SS	SS	LL	LL	LL	SS	LL	LL	SS	LL
9	rs3676086	SS	SS	LL	LL	LL	LL	LL	LL	SS	LL
9	rs13480166	SS	SS	LL	LL	LL	LL	LL	LL	SS	LL
9	gnf09.044.276	SS	SS	LL	LL	LL	LL	LL	LL	SS	SS
9	rs13480191	SS	SS	LL	LL	LL	SS	LL	LL	SS	SS

9	rs8259443	SS	SS	LL	LL	LL	SS	LL	LL	SS	SS
9	rs13480218	SS	SS	LL	LL	LL	LL	LL	LL	SS	SS
9	rs13480227	SS	SS	LL	LL	LL	LL	LL	LL	SS	SS
9	rs13480247	SS	SS	LL	SS	LL	LL	LL	LL	SS	SS
9	rs13480258	SS	SS	LL	SS	LL	LL	LL	LL	SS	SS
9	rs3703045	SS	SS	LL	SS	LL	LL	LL	SS	LL	SS
9	rs3724833	SS	SS	LL	LL	LL	LL	LL	SS	LL	SS
9	rs3670195	SS	SS	LL	LL	LL	LL	LL	SS	LL	SS
9	rs13480318	SS	SS	LL	SS	LL	LL	SS	SS	LL	SS
9	gnf09.087.298	SS	SS	LL	SS	LL	LL	LL	SS	LL	SS
9	rs13480387	SS	SS	LL	SS	LL	LL	LL	SS	LL	SS
9	rs3711089	SS	SS	LL	SS	LL	LL	LL	SS	LL	SS
9	rs6320810	SS	LL	SS	SS	LL	LL	LL	LL	SS	SS
9	rs3669563	SS	LL	SS	SS	LL	LL	LL	LL	SS	SS
9	rs13480454	SS	LL	SS	SS	LL	LL	LL	LL	SS	SS
9	rs6299531	SS	LL	SS	SS	LL	LL	LL	LL	LL	SS
9	rs8241505	SS	LL	SS	SS	LL	LL	LL	LL	LL	SS
10	rs13480510	LL	LL	LL	SS	LL	SS	SS	LL	LL	SS
10	rs13480516	LL	LL	LL	SS	LL	SS	SS	LL	LL	SS
10	rs13480527	LL	LL	LL	SS	LL	SS	SS	LL	LL	SS
10	rs3679120	LL	SS	LL	SS	LL	SS	SS	LL	LL	SS
10	rs13480547	LL	SS	LL	SS	LL	SS	SS	LL	LL	SS
10	rs13480601	SS	SS	LL	LL	SS	LL	SS	LL	LL	SS
10	rs3715820	SS	SS	LL	LL	LL	LL	SS	LL	LL	SS
10	rs6186864	SS	SS	LL	LL	SS	LL	SS	LL	LL	SS
10	rs13480630	LL	SS	LL	LL	SS	LL	SS	LL	LL	SS
10	rs13480638	LL	SS	LL	LL	LL	LL	SS	LL	SS	SS
10	rs13480652	LL	LL	LL	LL	LL	LL	SS	LL	SS	SS
10	rs13480678	SS	LL	LL	LL	LL	LL	SS	SS	SS	SS
10	mCV25373751	SS	LL	LL	LL	LL	LL	SS	SS	SS	LL
10	rs3661495	SS	LL	LL	LL	LL	LL	SS	SS	SS	LL
10	rs3704401	SS	SS	LL	LL	LL	LL	SS	SS	SS	LL
10	rs13480739	SS	SS	LL	LL	LL	LL	SS	SS	SS	LL
10	rs13480752	SS	SS	LL	SS	LL	SS	SS	SS	SS	LL
10	rs4228452	SS	SS	LL	SS	LL	SS	SS	SS	SS	LL
10	rs13480773	LL	SS	LL	SS	LL	SS	SS	SS	SS	LL
10	CZECH-10_116791624	LL	SS	LL	SS	LL	SS	SS	SS	SS	LL
10	rs6335076	LL	SS	LL	SS	LL	SS	SS	SS	SS	LL
10	rs6317716	LL	SS	LL	SS	LL	SS	SS	LL	SS	LL
10	rs13480808	LL	SS	LL	SS	LL	SS	LL	LL	LL	LL
11	rs13480835	SS	LL	LL	LL	LL	LL	SS	LL	LL	SS
11	rs6393401	LL	LL	LL	LL	LL	LL	SS	LL	LL	SS

11	rs13480853	LL	LL	LL	LL	LL	LL	SS	LL	SS	SS
11	rs3689494	LL	LL	LL	LL	LL	LL	SS	LL	LL	SS
11	rs3658216	LL	LL	LL	LL	LL	LL	SS	LL	SS	SS
11	rs13480913	LL	LL	LL	LL	LL	LL	SS	LL	SS	SS
11	rs13480918	LL	LL	SS	LL	LL	LL	SS	LL	SS	SS
11	UT_11_29.443258	LL	LL	SS	SS	LL	LL	SS	LL	SS	SS
11	rs3690160	LL	LL	SS	LL	LL	LL	SS	SS	LL	SS
11	rs3660692	LL	LL	SS	SS	LL	SS	SS	SS	LL	SS
11	rs6326787	LL	LL	SS	SS	LL	SS	SS	SS	LL	SS
11	rs6199956	LL	LL	LL	SS	LL	SS	SS	SS	LL	SS
11	rs13481031	LL	LL	LL	LL	LL	SS	SS	SS	LL	SS
11	rs3684076	SS	LL	LL	LL	LL	SS	SS	SS	LL	SS
11	rs13481050	SS	SS	LL	LL	SS	SS	SS	SS	LL	SS
11	rs3714311	LL	SS	LL	LL	SS	SS	SS	LL	LL	SS
11	rs13481075	LL	LL	LL	LL	SS	SS	SS	LL	LL	SS
11	rs13481093	LL	LL	LL	LL	SS	SS	SS	SS	LL	SS
11	CEL-11_73459270	LL	LL	LL	LL	SS	LL	SS	SS	LL	SS
11	rs6178421	SS	LL	LL	LL	SS	LL	SS	SS	LL	SS
11	rs13481123	SS	LL	LL	LL	SS	LL	SS	SS	LL	SS
11	rs3661657	SS	LL	LL	LL	SS	LL	SS	SS	LL	SS
11	rs3688955	SS	LL	LL	LL	SS	LL	SS	SS	LL	SS
11	rs13481161	SS	LL	LL	LL	SS	LL	SS	SS	LL	SS
11	rs3686162	SS	LL	LL	LL	SS	LL	SS	SS	LL	SS
11	rs13474433	SS	LL	LL	SS	SS	LL	SS	SS	LL	SS
11	rs3695865	SS	LL	LL	SS	SS	LL	SS	SS	LL	SS
11	rs6180460	SS	LL	LL	SS	LL	LL	SS	SS	LL	SS
11	rs6407687	SS	LL	LL	SS	LL	LL	SS	SS	LL	SS
11	rs13481210	SS	LL	LL	SS	SS	LL	SS	SS	LL	SS
11	rs13481220	SS	LL	LL	SS	SS	LL	SS	SS	SS	SS
11	rs13481228	SS	LL	LL	SS	SS	SS	SS	SS	SS	SS
11	rs13481233	SS	LL	LL	SS	SS	LL	SS	SS	SS	SS
11	gnf11.121.400	LL	LL	LL	SS	SS	LL	SS	SS	LL	SS
11	rs3662930	LL	LL	LL	SS	SS	LL	SS	SS	LL	SS
11	gnf11.125.992	LL	LL	LL	SS	LL	LL	SS	SS	LL	SS
11	CEL-11_118234030	LL	LL	LL	SS	SS	LL	SS	SS	LL	SS
12	rs3699421	SS	SS	LL	LL	LL	LL	LL	LL	SS	SS
12	rs13481308	SS	SS	LL	LL	LL	LL	LL	LL	SS	SS
12	rs6328018	SS	SS	LL	LL	LL	LL	LL	LL	SS	SS
12	UT_12_24.561109	SS	SS	LL	LL	LL	LL	LL	LL	SS	SS
12	rs3695382	SS	SS	LL	LL	LL	LL	LL	LL	SS	SS
12	rs13481408	SS	SS	LL	LL	SS	LL	LL	LL	SS	LL
12	rs6317361	SS	SS	LL	LL	SS	LL	SS	SS	SS	LL

12	gnf12.053.286	SS	SS	LL	LL	SS	SS	SS	SS	SS	LL
12	rs3660822	SS	SS	LL	LL	SS	SS	SS	SS	SS	LL
12	rs6335879	SS	SS	LL	LL	SS	SS	SS	SS	SS	LL
12	rs3687032	SS	SS	LL	LL	SS	SS	SS	SS	LL	LL
12	rs3709008	SS	SS	LL	LL	SS	SS	LL	SS	LL	LL
12	rs3682382	SS	SS	LL	LL	SS	SS	LL	SS	LL	LL
12	rs3654718	SS	LL	LL	LL	SS	SS	LL	SS	LL	LL
12	rs13481561	SS	LL	LL	LL	SS	SS	SS	SS	LL	LL
12	rs6263380	SS	LL	LL	LL	SS	LL	SS	SS	LL	LL
12	rs13481571	SS	LL	LL	LL	LL	SS	SS	SS	SS	SS
12	rs3711162	LL	LL	LL	LL	LL	SS	SS	SS	SS	SS
12	rs6288403	LL	LL	LL	LL	LL	LL	SS	SS	SS	SS
12	rs13481592	LL	LL	LL	SS	SS	LL	SS	SS	SS	SS
12	rs3679514	LL	LL	LL	SS	SS	LL	LL	SS	SS	SS
12	rs13481618	LL	LL	LL	SS	SS	LL	LL	SS	SS	SS
12	rs13481632	LL	LL	LL	LL	SS	LL	LL	SS	SS	LL
12	rs13481641	LL	LL	LL	LL	SS	LL	LL	SS	SS	LL
12	rs13481651	LL	LL	LL	LL	SS	LL	LL	SS	SS	LL
12	rs4229612	LL	LL	LL	SS	LL	SS	LL	SS	SS	LL
13	rs6215262	LL	SS	SS	SS	SS	LL	SS	SS	SS	LL
13	rs13481702	LL	SS	SS	SS	SS	SS	SS	SS	SS	LL
13	rs13481706	LL	SS	SS	SS	SS	SS	SS	SS	LL	LL
13	rs3678616	LL	SS	SS	SS	SS	SS	SS	SS	LL	LL
13	gnf13.016.175	LL	SS	SS	SS	SS	SS	SS	SS	LL	LL
13	rs4229685	LL	SS	LL	SS	SS	SS	SS	SS	SS	SS
13	rs3724709	LL	SS	LL	SS	LL	SS	SS	SS	SS	SS
13	gnf13.038.133	SS	SS	LL	SS	LL	SS	SS	SS	SS	SS
13	rs6271232	SS	SS	LL	LL	LL	SS	SS	SS	SS	SS
13	rs3688207	SS	SS	LL	LL	LL	SS	SS	SS	SS	SS
13	rs13481823	SS	SS	LL	LL	LL	SS	SS	LL	SS	SS
13	gnf13.057.762	LL	SS	LL	LL	LL	SS	SS	LL	SS	SS
13	rs6410679	LL	SS	LL	LL	LL	SS	SS	LL	LL	SS
13	rs6381045	LL	SS	LL	LL	LL	SS	SS	LL	LL	SS
13	rs3721965	LL	SS	LL	LL	LL	SS	SS	LL	LL	SS
13	rs13481886	LL	SS	LL	LL	SS	SS	SS	LL	LL	SS
13	rs13481892	LL	SS	LL	LL	SS	LL	SS	LL	LL	SS
13	mCV24625340	LL	SS	LL	LL	SS	LL	SS	LL	LL	LL
13	gnf13.092.499	LL	SS	SS	LL	SS	LL	SS	LL	LL	LL
13	rs13481958	LL	SS	SS	LL	SS	LL	SS	LL	LL	LL
13	rs3688959	LL	SS	SS	LL	LL	LL	SS	SS	LL	LL
13	rs6389588	LL	SS	LL	LL	LL	LL	SS	SS	LL	LL
13	rs13482000	LL	SS	LL	LL	LL	LL	SS	SS	LL	LL

13	mCV24886326	LL	SS	LL	SS	LL	LL	SS	SS	SS	LL
13	rs6247696	LL	SS	LL	SS	LL	SS	SS	SS	SS	LL
13	rs6397687	LL	LL	LL	SS	LL	SS	SS	SS	SS	LL
14	rs3687889	SS	LL	LL	LL	LL	LL	SS	SS	SS	SS
14	rs6397486	SS	LL	LL	LL	LL	LL	SS	SS	SS	SS
14	mCV23128760	SS	LL	LL	LL	LL	LL	SS	LL	SS	SS
14	rs3701623	SS	LL	LL	LL	LL	LL	SS	LL	SS	SS
14	rs13482262	SS	LL	LL	LL	LL	LL	SS	SS	SS	SS
14	rs6352085	SS	LL	LL	LL	LL	LL	SS	SS	SS	SS
14	rs13482276	SS	LL	LL	LL	LL	LL	SS	SS	SS	SS
14	rs13482281	SS	LL	LL	LL	LL	LL	SS	SS	SS	SS
14	rs13482296	SS	LL	LL	SS	LL	SS	SS	SS	SS	SS
14	rs6211694	SS	LL	LL	SS	LL	SS	SS	LL	SS	SS
14	CEL-14_110783830	SS	LL	LL	SS	LL	SS	SS	LL	SS	SS
14	rs3707842	SS	LL	LL	SS	LL	SS	SS	LL	SS	SS
14	gnf14.117.278	SS	LL	LL	SS	LL	SS	SS	LL	SS	SS
14	rs13482416	SS	LL	LL	SS	LL	SS	SS	LL	SS	SS
15	rs13459176	SS	LL	LL	LL	LL	LL	LL	SS	LL	LL
15	rs13482431	SS	LL	LL	LL	LL	LL	LL	SS	LL	LL
15	rs13482446	SS	LL	SS	LL	LL	LL	LL	SS	LL	SS
15	CEL-15_15919629	SS	SS	SS	LL	LL	LL	LL	SS	LL	SS
15	rs13482461	SS	SS	SS	LL	LL	SS	LL	SS	LL	SS
15	rs13482486	SS	SS	SS	LL	LL	SS	LL	SS	LL	SS
15	rs3670581	SS	SS	SS	LL	LL	SS	SS	SS	LL	SS
15	rs13482498	SS	SS	SS	SS	LL	SS	SS	SS	LL	SS
15	rs3088525	SS	SS	SS	SS	LL	SS	SS	SS	LL	SS
15	rs6188239	SS	SS	SS	LL	LL	SS	SS	SS	LL	SS
15	rs3695416	SS	LL	SS	LL	LL	SS	SS	SS	LL	SS
15	rs3677296	SS	LL	SS	LL	LL	SS	SS	SS	LL	SS
15	rs13482536	SS	LL	SS	SS	LL	LL	SS	SS	SS	SS
15	rs13482541	SS	LL	SS	LL	LL	LL	SS	SS	SS	SS
15	rs3660290	SS	LL	SS	LL	LL	LL	SS	SS	SS	SS
15	rs13482589	SS	SS	SS	LL	LL	LL	SS	SS	SS	SS
15	rs13482595	SS	SS	SS	LL	LL	LL	LL	SS	SS	SS
15	rs13482612	SS	SS	SS	LL	LL	LL	SS	SS	LL	SS
15	rs13482618	SS	SS	SS	LL	LL	LL	SS	SS	LL	SS
15	rs6197332	SS	SS	SS	LL	LL	LL	SS	SS	LL	SS
15	rs13482642	SS	SS	SS	SS	LL	LL	SS	SS	LL	SS
15	rs6287697	LL	SS	SS	SS	LL	LL	SS	LL	LL	SS
15	rs13482711	SS	SS	SS	SS	LL	LL	SS	LL	LL	SS
15	rs13482719	SS	SS	LL	SS	LL	LL	SS	LL	LL	SS
15	rs13482726	SS	SS	LL	SS	LL	SS	SS	LL	LL	SS

15	rs13482732	SS	SS	SS	SS	LL	SS	SS	LL	LL	SS
15	rs3708604	SS	SS	SS	LL	LL	SS	LL	LL	LL	SS
15	rs13482741	SS	SS	SS	LL	LL	LL	LL	LL	LL	SS
16	rs4152386	LL	SS	LL	SS	SS	SS	SS	LL	SS	SS
16	rs4165119	LL	SS	LL	LL	SS	LL	SS	LL	SS	LL
16	rs6294027	LL	SS	LL	LL	SS	LL	LL	LL	SS	LL
16	rs4172338	LL	SS	LL	SS	SS	SS	LL	LL	SS	LL
16	rs4173709	LL	SS	SS	SS	SS	SS	LL	LL	SS	LL
16	rs4175608	LL	SS	SS	SS	SS	SS	LL	LL	SS	LL
16	rs4177203	LL	SS	SS	SS	SS	SS	LL	LL	SS	LL
16	rs4179117	SS	SS	SS	SS	SS	SS	SS	LL	SS	SS
16	rs4184315	SS	SS	SS	SS	LL	SS	SS	LL	SS	SS
16	rs4185176	SS	SS	SS	SS	LL	SS	SS	LL	SS	SS
16	rs6271301	SS	SS	SS	SS	SS	SS	SS	LL	SS	SS
16	rs4192132	SS	SS	SS	SS	SS	SS	SS	LL	SS	LL
16	rs4197416	SS	SS	SS	SS	SS	SS	SS	LL	SS	LL
16	rs4197725	SS	LL	SS	SS	SS	SS	SS	LL	SS	LL
16	rs4202372	SS	LL	SS	SS	SS	SS	SS	LL	SS	LL
16	rs4203891	SS	SS	SS	SS	SS	LL	SS	LL	SS	LL
16	rs4205524	SS	SS	SS	SS	SS	LL	SS	LL	SS	LL
16	rs4211515	SS	SS	SS	SS	SS	LL	SS	LL	SS	LL
16	rs4211731	SS	SS	SS	SS	SS	SS	SS	LL	SS	LL
16	rs4219239	SS	SS	LL	SS	SS	SS	SS	LL	SS	LL
16	rs4219897	SS	SS	LL	SS	SS	SS	SS	LL	SS	SS
17	rs3724616	LL	SS	LL	LL	LL	LL	LL	SS	SS	LL
17	rs3702484	SS	SS	LL	LL	LL	LL	SS	SS	SS	LL
17	rs4231344	SS	SS	LL	LL	SS	LL	SS	SS	SS	LL
17	rs3090500	SS	SS	LL	SS	SS	LL	SS	SS	SS	LL
17	rs4136360	SS	SS	LL	SS	SS	NA	SS	SS	SS	LL
17	rs6358703	SS	LL	LL	SS	SS	NA	SS	SS	SS	LL
17	rs13482947	LL	LL	LL	SS	LL	NA	SS	SS	SS	LL
17	gnf17.035.152	LL	LL	LL	SS	LL	NA	SS	SS	SS	LL
17	rs13482968	LL	LL	LL	LL	LL	NA	SS	SS	SS	LL
17	rs13482973	LL	LL	LL	LL	LL	NA	SS	SS	SS	LL
17	rs3090988	LL	LL	LL	LL	LL	LL	SS	SS	SS	LL
17	mCV22888090	LL	LL	LL	SS	LL	LL	LL	LL	SS	LL
17	rs13483075	LL	LL	LL	SS	LL	SS	LL	LL	SS	LL
17	rs6322076	LL	LL	SS	SS	LL	LL	LL	LL	SS	LL
17	rs6386440	LL	LL	SS	SS	LL	LL	LL	LL	SS	SS
17	rs3684732	LL	SS	SS	LL	SS	SS	SS	LL	SS	SS
17	rs3701338	LL	SS	SS	LL	SS	SS	LL	LL	SS	SS
17	rs13483144	LL	SS	SS	LL	SS	SS	LL	LL	SS	SS

18	rs13483183	LL	LL	SS	LL	LL	LL	SS	LL	SS	LL
18	rs13483200	SS	LL	SS	LL	LL	LL	SS	LL	SS	LL
18	rs13483210	SS	LL	SS	LL	LL	LL	SS	LL	SS	LL
18	mCV23617245	LL	LL	SS	LL	LL	LL	SS	LL	SS	LL
18	rs6194744	LL	LL	LL	LL	LL	LL	SS	LL	SS	LL
18	rs13483262	LL	LL	LL	LL	LL	LL	SS	SS	SS	LL
18	rs13483277	LL	LL	LL	LL	LL	LL	SS	SS	LL	LL
18	CEL-18_32158369	LL	LL	LL	LL	LL	LL	SS	SS	LL	LL
18	rs3675531	LL	LL	SS	LL	LL	LL	SS	SS	LL	LL
18	gnf18.033.953	LL	LL	SS	LL	SS	LL	SS	SS	LL	LL
18	rs13483319	LL	LL	SS	LL	SS	LL	SS	SS	LL	SS
18	rs6313313	LL	LL	SS	SS	SS	SS	SS	SS	LL	SS
18	rs3676196	SS	LL	SS	SS	SS	SS	SS	SS	LL	SS
18	rs13483340	SS	LL	SS	SS	SS	SS	SS	SS	LL	SS
18	rs6184541	SS	LL	LL	SS	SS	SS	SS	SS	LL	SS
18	rs3654438	SS	LL	LL	SS	SS	SS	SS	SS	LL	SS
18	rs3688789	SS	LL	LL	SS	SS	SS	SS	LL	LL	SS
18	rs3716803	SS	LL	LL	SS	SS	SS	LL	LL	LL	SS
18	rs13483423	SS	LL	LL	SS	SS	SS	LL	SS	LL	SS
18	gnf18.069.928	SS	LL	LL	SS	SS	SS	SS	SS	LL	SS
18	rs6302629	SS	LL	LL	SS	LL	SS	SS	SS	LL	SS
18	rs3705890	SS	LL	LL	SS	LL	SS	SS	SS	LL	SS
18	rs3671707	LL	LL	LL	SS	LL	SS	LL	SS	LL	SS
18	rs13483466	LL	LL	LL	SS	SS	SS	LL	SS	LL	SS
19	rs3713033	LL	LL	SS	LL	SS	LL	LL	LL	SS	LL
19	rs13483526	LL	LL	SS	LL	SS	LL	LL	LL	SS	LL
19	rs6316813	LL	LL	SS	SS	SS	SS	LL	LL	SS	LL
19	rs6307076	LL	LL	SS	SS	SS	SS	LL	LL	SS	LL
19	rs6342493	SS	LL	SS	SS	SS	SS	LL	LL	LL	SS
19	rs6291559	SS	LL	SS	SS	SS	SS	LL	LL	LL	LL
19	rs3714482	SS	LL	LL	SS	SS	SS	LL	LL	LL	LL
19	CEL-19_32349880	SS	LL	SS	SS	SS	SS	LL	LL	SS	SS
19	rs13483650	SS	LL	SS	SS	LL	SS	LL	SS	LL	SS
19	CEL-19_48242857	SS	LL	SS	SS	LL	SS	LL	SS	LL	SS
19	rs13483677	SS	LL	SS	SS	LL	SS	LL	SS	LL	SS
19	rs3711945	SS	LL	SS	SS	SS	SS	LL	SS	LL	SS
X	rs13483712	LL	SS	LL	LL	SS	LL	LL	LL	SS	LL
X	rs13483724	LL	SS	LL	LL	LL	LL	LL	LL	SS	LL
X	rs13483777	LL	SS	LL	LL	LL	LL	SS	LL	SS	LL
X	rs13483884	LL	SS	LL	LL	LL	LL	SS	LL	SS	LL
X	rs13483899	SS	SS	LL	LL	LL	LL	SS	LL	SS	LL
X	gnfX.084.751	SS	SS	LL	LL	LL	LL	SS	LL	SS	LL

X	gnfX.086.039	SS	SS	LL	LL	LL	LL	SS	LL	SS	LL
X	rs13483951	SS	SS	LL	SS	LL	LL	SS	LL	SS	LL
X	rs6205221	SS	LL	LL	SS	LL	LL	SS	LL	SS	LL
X	rs13484003	SS	LL	LL	LL	LL	LL	SS	LL	SS	LL
X	gnfX.118.600	SS	LL	LL	LL	LL	LL	SS	LL	SS	LL
X	rs13484038	SS	LL	LL	LL	LL	LL	SS	LL	LL	LL
X	gnfX.124.190	SS	LL	LL	LL	LL	LL	SS	LL	SS	LL
X	CEL-X_133525088	SS	SS	LL	LL	LL	LL	SS	LL	SS	LL
X	rs13484050	SS	SS	LL	LL	LL	LL	SS	LL	SS	LL
X	rs13484093	SS	SS	LL	LL	LL	LL	LL	LL	LL	LL
X	rs13484094	SS	SS	LL	LL	LL	LL	LL	LL	LL	LL
