# Supplementary material for: Quantitative Trait Loci Affecting Liver Fat Content in Mice
Source: G3 (Bethesda). 2012 Sep 1;2(9):1019–25. doi: 10.1534/g3.112.003343 (PMC3429915; doi:10.1534/g3.112.003343)
Supplement: Supporting Information [file supp_2.9.1019_TableS1.pdf]

Table S1 **Number of mice (N), average liver fat content and standard deviation (SD), average liver fattiness and standard deviation (SD), by cohort.**

		Liver Fat Content		Liver Fattiness	
**Cohort**	**N**	**Average (%)**	**SD**	**Average (mg)**	**SD**
**4 All**	**21**	**0.30**	**0.04**	**1.02**	**1.39**
4 HF F	3	0.37	0.02	1.02	0.99
4 HF M	5	0.29	0.03	0.96	0.89
4 LF F	6	0.26	0.02	0.10	1.23
4 LF M	7	0.30	0.04	1.84	1.63
**5 All**	**28**	**0.26**	**0.05**	**-0.04**	**1.77**
5 HF F	7	0.29	0.04	0.65	1.67
5 HF M	9	0.24	0.04	-0.65	1.48
5 LF F	7	0.27	0.05	-0.55	2.21
5 LF M	5	0.26	0.03	0.79	1.55
**10 All**	**26**	**0.25**	**0.06**	**-0.43**	**2.53**
10 HF F	5	0.34	0.03	1.40	1.96
10 HF M	7	0.25	0.05	-0.06	2.21
10 LF F	6	0.26	0.02	0.61	0.75
10 LF M	8	0.19	0.04	-2.68	2.60
**15 All**	**31**	**0.22**	**0.05**	**-1.27**	**1.94**
15 HF F	6	0.23	0.05	-2.15	2.75
15 HF M	9	0.21	0.05	-1.64	2.19
15 LF F	9	0.24	0.04	-1.25	1.40
15 LF M	7	0.20	0.02	-0.20	1.09
**18 All**	**28**	**0.29**	**0.07**	**0.94**	**2.18**
18 HF F	6	0.31	0.03	1.22	0.99
18 HF M	7	0.35	0.07	3.05	1.76
18 LF F	9	0.23	0.03	-1.02	1.28
18 LF M	6	0.29	0.08	1.15	2.23
**19 All**	**31**	**0.26**	**0.04**	**0.41**	**1.54**
19HF F	7	0.30	0.05	1.20	0.40
19 HF M	8	0.26	0.02	0.07	1.62
19 LF F	7	0.26	0.04	0.05	1.12
19 LF M	9	0.24	0.05	0.35	2.19

**20 All**	**16**	**0.25**	**0.13**	**-1.12**	**2.50**
20 HF F	2	0.22	0.00	-1.32	0.89
20 HF M	4	0.24	0.19	-1.95	1.55
20 LF F	5	0.26	0.14	-1.66	2.10
20 LF M	5	0.25	0.12	0.17	3.74
**22 All**	**44**	**0.37**	**0.10**	**2.30**	**2.44**
22 HF F	12	0.41	0.06	3.42	1.82
22 HF M	12	0.46	0.05	3.55	2.25
22 LF F	12	0.32	0.06	1.00	2.63
22 LF M	8	0.26	0.08	0.69	1.38
**23 All**	**38**	**0.26**	**0.06**	**0.48**	**2.03**
23 HF F	7	0.31	0.07	1.72	2.71
23 HF M	10	0.25	0.06	-0.02	1.50
23 LF F	9	0.25	0.05	-0.48	1.73
23 LF M	12	0.24	0.04	0.89	1.94
**31 All**	**11**	**0.26**	**0.04**	**0.19**	**2.34**
31 HF F	4	0.27	0.05	-0.21	2.17
31 HF M	5	0.26	0.05	1.19	1.37
31 LF F	1	0.19	--	-5.26	--
31 LF M	2	0.28	0.01	1.22	1.04
**33 All**	**23**	**0.28**	**0.08**	**0.74**	**1.79**
33 HF F	4	0.40	0.09	3.20	0.83
33 HF M	7	0.28	0.05	0.65	1.20
33 LF F	7	0.23	0.03	-0.33	0.98
33 LF M	5	0.23	0.02	0.39	2.27
**35 All**	**26**	**0.25**	**0.07**	**-0.16**	**1.60**
35 HF F	6	0.26	0.04	-0.45	1.50
35 HF M	5	0.36	0.09	0.88	2.12
35 LF F	7	0.20	0.03	-1.07	1.34
35 LF M	8	0.23	0.04	0.08	1.11

**38 All**	**30**	**0.21**	**0.05**	**-1.43**	**1.95**
38 HF F	7	0.25	0.04	-2.64	3.15
38 HF M	7	0.18	0.04	-0.95	1.33
38 LF F	8	0.23	0.04	-0.78	1.21
38 LF M	8	0.17	0.03	-1.44	1.45
**45 All**	**16**	**0.29**	**0.03**	**0.64**	**2.85**
45 HF F	5	0.29	0.03	0.05	3.60
45 HF M	3	0.31	0.02	2.72	0.67
45 LF F	5	0.27	0.04	-1.16	2.58
45 LF M	3	0.32	0.02	2.51	0.72
**46 All**	**27**	**0.23**	**0.06**	**-0.40**	**2.14**
46 HF F	5	0.23	0.09	-1.00	3.73
46 HF M	6	0.24	0.07	0.29	1.18
46 LF F	8	0.21	0.04	-1.15	1.59
46 LF M	8	0.24	0.05	0.21	1.96
**48 All**	**28**	**0.31**	**0.11**	**1.14**	**3.19**
48 HF F	6	0.37	0.10	2.09	4.15
48 HF M	7	0.43	0.05	4.40	1.41
48 LF F	9	0.22	0.06	-0.92	2.02
48 LF M	6	0.22	0.05	-0.52	1.38
**LG All**	**25**	**0.19**	**0.05**	**-3.60**	**2.97**
LG HF F	6	0.24	0.02	-4.63	3.32
LG HF M	7	0.22	0.03	-2.13	2.32
LG LF F	6	0.15	0.03	-3.36	4.09
LG LF M	6	0.13	0.02	-4.27	1.74
**SM All**	**29**	**0.25**	**0.11**	**-0.94**	**2.48**
SM HF F	6	0.33	0.05	-0.18	1.97
SM HF M	11	0.33	0.10	0.41	2.51
SM LF F	6	0.14	0.02	-2.58	2.20
SM LF M	6	0.14	0.02	-2.52	1.50
