# Supplementary material for: Quantitative Trait Loci Affecting Liver Fat Content in Mice
Source: G3 (Bethesda). 2012 Sep 1;2(9):1019–25. doi: 10.1534/g3.112.003343 (PMC3429915; doi:10.1534/g3.112.003343)
Supplement: Supporting Information [file supp_2.9.1019_TableS2.pdf]

Table S2 Hepatic fat phenotypes.

	Sex	Diet	Strain	Fattiness	Fat Content
1	♀	LF	SM/J	-4.89	0.17
2	♀	LF	SM/J	-4.76	0.12
3	♂	LF	SM/J	-4.48	0.11
4	♂	LF	SM/J	-3.87	0.11
5	♂	HF	SM/J	-3.86	0.16
6	♀	LF	SM/J	-3.34	0.14
7	♂	LF	SM/J	-2.55	0.13
8	♀	LF	SM/J	-2.50	0.16
9	♀	HF	SM/J	-2.33	0.32
10	♂	LF	SM/J	-2.02	0.17
11	♂	LF	SM/J	-1.91	0.15
12	♀	HF	SM/J	-1.55	0.31
13	♂	HF	SM/J	-1.52	0.25
14	♀	HF	SM/J	-1.37	0.29
15	♂	HF	SM/J	-0.86	0.29
16	♂	HF	SM/J	-0.83	0.15
17	♂	HF	SM/J	-0.54	0.37
18	♀	LF	SM/J	-0.29	0.14
19	♂	LF	SM/J	-0.29	0.15
20	♀	HF	SM/J	-0.03	0.28
21	♂	HF	SM/J	0.15	0.41
22	♀	LF	SM/J	0.31	0.14
23	♂	HF	SM/J	0.85	0.34
24	♂	HF	SM/J	0.87	0.45
25	♂	HF	SM/J	0.97	0.37
26	♀	HF	SM/J	1.39	0.39
27	♀	HF	SM/J	2.81	0.37
28	♂	HF	SM/J	4.20	0.43
29	♂	HF	SM/J	5.06	0.40
30	♀	LF	LG/J	-9.99	0.14
31	♀	HF	LG/J	-8.94	0.24
32	♀	HF	LG/J	-7.72	0.23
33	♂	LF	LG/J	-6.71	0.15
34	♀	LF	LG/J	-6.05	0.12
35	♀	HF	LG/J	-5.33	0.23
36	♂	LF	LG/J	-5.26	0.12
37	♂	LF	LG/J	-5.21	0.11
38	♂	HF	LG/J	-4.81	0.25

39	♂	HF	LG/J	-4.31	0.17
40	♀	LF	LG/J	-3.82	0.12
41	♂	LF	LG/J	-3.61	0.14
42	♀	HF	LG/J	-3.50	0.27
43	♂	HF	LG/J	-3.03	0.20
44	♂	LF	LG/J	-2.57	0.16
45	♂	LF	LG/J	-2.28	0.13
46	♀	HF	LG/J	-1.86	0.26
47	♂	HF	LG/J	-1.19	0.21
48	♂	HF	LG/J	-0.57	0.23
49	♀	HF	LG/J	-0.42	0.23
50	♀	LF	LG/J	-0.17	0.16
51	♀	LF	LG/J	-0.11	0.19
52	♀	LF	LG/J	0.01	0.16
53	♂	HF	LG/J	1.15	0.22
54	♂	LF	4	-1.31	0.24
55	♀	LF	4	-1.27	0.23
56	♀	LF	4	-1.13	0.25
57	♀	LF	4	0.02	0.29
58	♂	HF	4	0.12	0.26
59	♂	HF	4	0.12	0.27
60	♀	LF	4	0.13	0.26
61	♀	HF	4	0.16	0.35
62	♀	HF	4	0.79	0.37
63	♂	HF	4	0.87	0.27
64	♀	LF	4	0.92	0.28
65	♂	LF	4	1.33	0.33
66	♂	HF	4	1.51	0.33
67	♂	LF	4	1.81	0.33
68	♂	LF	4	1.84	0.35
69	♀	LF	4	1.97	0.27
70	♀	HF	4	2.11	0.40
71	♂	HF	4	2.16	0.32
72	♂	LF	4	2.29	0.28
73	♂	LF	4	3.21	0.29
74	♂	LF	4	3.75	0.29
75	♀	LF	5	-3.73	0.25
76	♂	HF	5	-2.64	0.22
77	♂	HF	5	-2.43	0.28
78	♀	LF	5	-1.79	0.23
79	♀	LF	5	-1.79	0.24
80	♂	HF	5	-1.37	0.26

81	♀	HF	5	-1.36	0.22
82	♂	LF	5	-1.01	0.22
83	♀	HF	5	-0.94	0.27
84	♂	HF	5	-0.93	0.20
85	♂	HF	5	-0.92	0.23
86	♀	HF	5	-0.86	0.27
87	♀	LF	5	-0.46	0.32
88	♂	LF	5	-0.42	0.30
89	♂	HF	5	-0.39	0.19
90	♂	HF	5	-0.14	0.20
91	♀	LF	5	-0.10	0.23
92	♂	LF	5	0.77	0.23
93	♀	LF	5	0.86	0.25
94	♀	HF	5	0.93	0.28
95	♂	HF	5	1.18	0.30
96	♂	HF	5	1.79	0.28
97	♂	LF	5	1.99	0.24
98	♀	HF	5	2.01	0.33
99	♀	HF	5	2.27	0.33
100	♀	HF	5	2.47	0.34
101	♂	LF	5	2.63	0.29
102	♀	LF	5	3.18	0.35
103	♂	LF	10	-6.86	0.16
104	♂	LF	10	-5.55	0.18
105	♂	LF	10	-3.55	0.15
106	♂	HF	10	-3.15	0.28
107	♂	LF	10	-2.87	0.20
108	♂	LF	10	-2.28	0.18
109	♂	HF	10	-1.41	0.17
110	♂	HF	10	-0.85	0.20
111	♂	HF	10	-0.84	0.25
112	♀	HF	10	-0.64	0.35
113	♂	LF	10	-0.52	0.26
114	♂	LF	10	-0.34	0.24
115	♀	LF	10	-0.06	0.25
116	♀	LF	10	0.03	0.24
117	♀	HF	10	0.21	0.38
118	♀	LF	10	0.37	0.26
119	♀	LF	10	0.39	0.30
120	♀	HF	10	0.44	0.30
121	♂	HF	10	0.50	0.24
122	♂	LF	10	0.57	0.18

123	♀	LF	10	0.99	0.27
124	♂	HF	10	1.74	0.28
125	♀	LF	10	1.94	0.27
126	♀	HF	10	3.48	0.33
127	♀	HF	10	3.54	0.35
128	♂	HF	10	3.58	0.32
129	♀	HF	15	-5.93	0.14
130	♂	HF	15	-5.72	0.15
131	♀	HF	15	-4.23	0.26
132	♀	LF	15	-3.77	0.18
133	♂	HF	15	-3.35	0.15
134	♀	HF	15	-3.22	0.18
135	♂	HF	15	-3.21	0.29
136	♀	LF	15	-2.86	0.30
137	♂	LF	15	-2.78	0.23
138	♂	HF	15	-2.01	0.15
139	♀	LF	15	-1.31	0.29
140	♂	HF	15	-1.26	0.17
141	♀	LF	15	-1.07	0.20
142	♀	HF	15	-0.97	0.26
143	♂	HF	15	-0.88	0.19
144	♀	LF	15	-0.88	0.23
145	♀	LF	15	-0.40	0.21
146	♂	LF	15	-0.29	0.19
147	♂	HF	15	-0.29	0.26
148	♂	LF	15	-0.23	0.18
149	♀	LF	15	0.03	0.24
150	♂	LF	15	0.13	0.20
151	♂	LF	15	0.18	0.21
152	♀	LF	15	0.22	0.25
153	♂	LF	15	0.28	0.20
154	♂	LF	15	0.38	0.19
155	♀	HF	15	0.51	0.26
156	♂	LF	15	0.71	0.28
157	♂	HF	15	0.74	0.25
158	♀	HF	15	0.93	0.26
159	♂	HF	15	1.21	0.26
160	♀	LF	18	-2.91	0.26
161	♀	LF	18	-2.54	0.20
162	♂	LF	18	-2.17	0.26
163	♀	LF	18	-1.85	0.22
164	♀	LF	18	-1.21	0.20

165	♀	LF	18	-1.10	0.25
166	♀	LF	18	-0.87	0.21
167	♀	LF	18	0.04	0.18
168	♀	HF	18	0.18	0.31
169	♀	HF	18	0.30	0.29
170	♂	HF	18	0.38	0.24
171	♂	LF	18	0.50	0.28
172	♀	HF	18	0.52	0.30
173	♀	LF	18	0.56	0.27
174	♀	LF	18	0.68	0.26
175	♂	LF	18	0.84	0.25
176	♂	LF	18	0.95	0.25
177	♂	HF	18	1.81	0.38
178	♀	HF	18	1.88	0.28
179	♀	HF	18	1.99	0.32
180	♂	LF	18	2.11	0.28
181	♀	HF	18	2.43	0.35
182	♂	HF	18	2.53	0.27
183	♂	HF	18	2.81	0.35
184	♂	HF	18	3.93	0.41
185	♂	HF	18	4.04	0.37
186	♂	LF	18	4.66	0.45
187	♂	HF	18	5.84	0.46
188	♂	HF	19	-3.57	0.23
189	♂	LF	19	-3.02	0.20
190	♀	LF	19	-1.75	0.25
191	♂	LF	19	-1.73	0.24
192	♂	LF	19	-1.37	0.21
193	♂	HF	19	-0.60	0.22
194	♀	LF	19	-0.56	0.23
195	♀	LF	19	-0.40	0.23
196	♀	LF	19	-0.16	0.28
197	♂	LF	19	-0.14	0.17
198	♂	LF	19	0.05	0.23
199	♂	HF	19	0.42	0.24
200	♂	HF	19	0.43	0.25
201	♂	HF	19	0.45	0.26
202	♀	HF	19	0.74	0.28
203	♂	HF	19	0.75	0.27
204	♀	LF	19	0.75	0.23
205	♀	HF	19	0.76	0.30
206	♂	HF	19	0.82	0.30

207	♀	LF	19	0.83	0.29
208	♀	HF	19	0.99	0.31
209	♂	LF	19	1.24	0.29
210	♀	HF	19	1.27	0.28
211	♀	HF	19	1.36	0.36
212	♀	HF	19	1.49	0.21
213	♀	LF	19	1.64	0.33
214	♀	HF	19	1.82	0.34
215	♂	HF	19	1.89	0.27
216	♂	LF	19	2.07	0.24
217	♂	LF	19	2.27	0.31
218	♂	LF	19	3.83	0.29
219	♀	LF	20	-4.30	0.18
220	♂	HF	20	-4.26	0.52
221	♂	LF	20	-3.70	0.18
222	♀	LF	20	-3.41	0.16
223	♂	LF	20	-3.27	0.15
224	♀	HF	20	-1.95	0.22
225	♂	HF	20	-1.43	0.15
226	♂	HF	20	-1.14	0.13
227	♂	HF	20	-0.99	0.15
228	♀	LF	20	-0.93	0.14
229	♀	HF	20	-0.69	0.22
230	♀	LF	20	-0.30	0.39
231	♂	LF	20	0.49	0.17
232	♀	LF	20	0.63	0.45
233	♂	LF	20	2.16	0.32
234	♂	LF	20	5.17	0.42
235	♀	LF	22	-4.83	0.32
236	♀	LF	22	-2.18	0.24
237	♂	LF	22	-2.00	0.12
238	♂	HF	22	-0.77	0.47
239	♀	LF	22	-0.74	0.28
240	♂	LF	22	-0.37	0.22
241	♀	HF	22	-0.25	0.28
242	♀	LF	22	-0.16	0.25
243	♂	HF	22	0.12	0.45
244	♀	LF	22	0.28	0.38
245	♂	LF	22	0.36	0.40
246	♂	LF	22	0.61	0.22
247	♀	HF	22	0.92	0.47
248	♂	LF	22	1.31	0.28

249	♂	LF	22	1.43	0.27
250	♀	LF	22	1.60	0.27
251	♀	HF	22	1.67	0.45
252	♂	LF	22	1.85	0.26
253	♂	HF	22	2.04	0.50
254	♀	LF	22	2.21	0.31
255	♂	LF	22	2.30	0.32
256	♀	LF	22	2.46	0.30
257	♀	LF	22	2.57	0.35
258	♂	HF	22	2.88	0.43
259	♀	HF	22	2.96	0.35
260	♂	HF	22	3.16	0.50
261	♀	LF	22	3.21	0.36
262	♀	HF	22	3.39	0.46
263	♂	HF	22	3.41	0.48
264	♀	HF	22	3.62	0.37
265	♂	HF	22	3.65	0.34
266	♀	LF	22	3.80	0.38
267	♀	LF	22	3.81	0.42
268	♀	HF	22	3.99	0.39
269	♀	HF	22	4.18	0.42
270	♀	HF	22	4.77	0.44
271	♀	HF	22	4.78	0.41
272	♂	HF	22	4.93	0.48
273	♂	HF	22	5.39	0.49
274	♀	HF	22	5.47	0.44
275	♀	HF	22	5.52	0.45
276	♂	HF	22	5.54	0.44
277	♂	HF	22	6.00	0.49
278	♂	HF	22	6.23	0.50
279	♀	HF	23	-2.82	0.27
280	♂	LF	23	-2.57	0.14
281	♂	HF	23	-2.48	0.25
282	♂	HF	23	-2.32	0.20
283	♀	LF	23	-2.10	0.19
284	♀	LF	23	-1.91	0.23
285	♀	LF	23	-1.86	0.32
286	♀	LF	23	-1.79	0.25
287	♀	LF	23	-1.63	0.17
288	♀	HF	23	-1.29	0.16
289	♂	LF	23	-0.92	0.20
290	♂	LF	23	-0.82	0.22

291	♂	LF	23	-0.51	0.22
292	♂	HF	23	-0.43	0.20
293	♂	HF	23	-0.27	0.17
294	♂	HF	23	0.11	0.28
295	♂	LF	23	0.13	0.23
296	♂	HF	23	0.18	0.37
297	♀	LF	23	0.58	0.28
298	♂	HF	23	0.61	0.25
299	♂	HF	23	0.71	0.20
300	♀	LF	23	0.79	0.28
301	♂	LF	23	0.82	0.24
302	♂	LF	23	0.84	0.25
303	♀	LF	23	1.13	0.28
304	♂	HF	23	1.17	0.28
305	♂	LF	23	1.46	0.28
306	♀	HF	23	2.00	0.30
307	♂	LF	23	2.43	0.28
308	♂	LF	23	2.45	0.24
309	♂	HF	23	2.46	0.31
310	♀	LF	23	2.52	0.27
311	♀	HF	23	2.70	0.32
312	♂	LF	23	3.46	0.29
313	♀	HF	23	3.53	0.36
314	♂	LF	23	3.88	0.28
315	♀	HF	23	3.91	0.37
316	♀	HF	23	4.03	0.36
317	♀	LF	31	-5.26	0.19
318	♀	HF	31	-3.34	0.31
319	♂	HF	31	-0.59	0.21
320	♀	HF	31	0.05	0.22
321	♂	LF	31	0.48	0.29
322	♂	HF	31	1.02	0.32
323	♂	HF	31	1.03	0.24
324	♀	HF	31	1.04	0.32
325	♂	HF	31	1.21	0.24
326	♀	HF	31	1.42	0.25
327	♂	LF	31	1.96	0.27
328	♂	HF	31	3.27	0.29
329	♂	LF	33	-2.79	0.21
330	♀	LF	33	-2.35	0.18
331	♂	HF	33	-1.71	0.18
332	♂	LF	33	-0.45	0.23

333	♀	LF	33	-0.34	0.25
334	♀	LF	33	-0.26	0.24
335	♀	LF	33	-0.23	0.25
336	♀	LF	33	-0.13	0.25
337	♀	LF	33	0.11	0.20
338	♂	LF	33	0.12	0.22
339	♂	HF	33	0.14	0.34
340	♂	HF	33	0.35	0.27
341	♀	LF	33	0.85	0.27
342	♂	HF	33	1.15	0.30
343	♂	HF	33	1.18	0.28
344	♂	HF	33	1.64	0.30
345	♂	HF	33	1.77	0.25
346	♂	LF	33	2.04	0.25
347	♀	HF	33	2.19	0.29
348	♀	HF	33	2.87	0.39
349	♂	LF	33	3.04	0.27
350	♀	HF	33	3.72	0.52
351	♀	HF	33	4.02	0.40
352	♀	LF	35	-3.02	0.16
353	♀	LF	35	-2.36	0.17
354	♂	LF	35	-2.26	0.29
355	♂	HF	35	-2.13	0.26
356	♀	HF	35	-2.02	0.26
357	♀	LF	35	-1.95	0.21
358	♀	HF	35	-1.41	0.27
359	♀	HF	35	-1.25	0.28
360	♀	HF	35	-0.70	0.19
361	♂	LF	35	-0.46	0.18
362	♂	HF	35	-0.45	0.46
363	♀	LF	35	-0.43	0.19
364	♀	LF	35	-0.21	0.22
365	♂	LF	35	0.03	0.21
366	♀	LF	35	0.05	0.22
367	♂	LF	35	0.06	0.19
368	♀	LF	35	0.41	0.22
369	♂	LF	35	0.43	0.23
370	♂	LF	35	0.51	0.24
371	♂	HF	35	0.70	0.44
372	♀	HF	35	0.73	0.30
373	♂	HF	35	0.88	0.27
374	♂	LF	35	1.01	0.23

375	♂	LF	35	1.34	0.28
376	♀	HF	35	1.94	0.27
377	♂	HF	35	2.26	0.26
378	♂	HF	35	3.99	0.36
379	♀	HF	38	-6.51	0.20
380	♀	HF	38	-5.45	0.25
381	♀	HF	38	-5.26	0.24
382	♂	HF	38	-3.57	0.13
383	♂	LF	38	-3.28	0.21
384	♂	LF	38	-3.18	0.15
385	♀	LF	38	-2.69	0.22
386	♀	HF	38	-2.67	0.23
387	♂	LF	38	-2.29	0.14
388	♀	LF	38	-2.00	0.20
389	♂	LF	38	-1.65	0.18
390	♂	HF	38	-1.28	0.13
391	♂	LF	38	-1.24	0.21
392	♂	HF	38	-1.03	0.16
393	♀	LF	38	-0.95	0.18
394	♀	LF	38	-0.76	0.30
395	♀	LF	38	-0.74	0.22
396	♂	HF	38	-0.64	0.21
397	♀	LF	38	-0.61	0.21
398	♂	HF	38	-0.51	0.21
399	♂	LF	38	-0.51	0.14
400	♂	HF	38	-0.41	0.22
401	♂	LF	38	-0.24	0.18
402	♀	HF	38	0.00	0.26
403	♀	LF	38	0.44	0.25
404	♀	HF	38	0.48	0.28
405	♂	HF	38	0.81	0.20
406	♂	LF	38	0.83	0.17
407	♀	HF	38	0.95	0.33
408	♀	LF	38	1.11	0.27
409	♀	LF	45	-5.40	0.24
410	♀	HF	45	-5.32	0.26
411	♀	HF	45	-1.70	0.29
412	♀	LF	45	-1.20	0.22
413	♀	LF	45	-0.41	0.28
414	♀	LF	45	-0.31	0.28
415	♀	HF	45	1.53	0.30
416	♀	LF	45	1.54	0.33

417	♂	HF	45	1.97	0.32
418	♀	HF	45	1.98	0.27
419	♂	LF	45	2.05	0.29
420	♂	LF	45	2.13	0.34
421	♂	HF	45	2.92	0.31
422	♂	HF	45	3.27	0.29
423	♂	LF	45	3.34	0.32
424	♀	HF	45	3.79	0.34
425	♀	HF	46	-6.92	0.19
426	♂	LF	46	-4.20	0.17
427	♀	LF	46	-3.88	0.16
428	♀	LF	46	-2.90	0.13
429	♀	LF	46	-2.04	0.21
430	♀	HF	46	-1.53	0.14
431	♂	HF	46	-1.33	0.18
432	♂	HF	46	-0.57	0.27
433	♀	LF	46	-0.56	0.19
434	♂	LF	46	-0.44	0.17
435	♀	LF	46	-0.32	0.25
436	♀	HF	46	-0.29	0.19
437	♂	HF	46	-0.28	0.18
438	♀	LF	46	0.02	0.22
439	♀	LF	46	0.05	0.26
440	♂	LF	46	0.09	0.19
441	♂	LF	46	0.17	0.23
442	♀	LF	46	0.46	0.23
443	♀	HF	46	0.56	0.31
444	♂	HF	46	0.93	0.17
445	♂	LF	46	1.28	0.30
446	♂	HF	46	1.44	0.29
447	♂	LF	46	1.54	0.26
448	♂	HF	46	1.55	0.33
449	♂	LF	46	1.55	0.28
450	♂	LF	46	1.68	0.28
451	♀	HF	46	3.17	0.34
452	♀	LF	48	-5.10	0.16
453	♀	HF	48	-4.85	0.20
454	♀	LF	48	-3.12	0.17
455	♂	LF	48	-2.75	0.16
456	♀	LF	48	-1.33	0.16
457	♀	LF	48	-1.17	0.18
458	♂	LF	48	-1.04	0.18

459	♂	LF	48	-0.65	0.25
460	♂	LF	48	-0.43	0.20
461	♀	HF	48	-0.08	0.31
462	♀	LF	48	0.04	0.25
463	♀	LF	48	0.50	0.29
464	♂	LF	48	0.53	0.21
465	♀	LF	48	0.55	0.26
466	♀	LF	48	0.64	0.23
467	♀	LF	48	0.66	0.31
468	♂	LF	48	1.24	0.30
469	♀	HF	48	2.12	0.39
470	♂	HF	48	2.97	0.35
471	♀	HF	48	3.17	0.41
472	♂	HF	48	3.43	0.40
473	♂	HF	48	3.57	0.45
474	♂	HF	48	3.98	0.46
475	♂	HF	48	4.14	0.41
476	♂	HF	48	5.94	0.46
477	♀	HF	48	5.95	0.43
478	♀	HF	48	6.23	0.48
479	♂	HF	48	6.78	0.50

♀ = female, ♂ = male, HF = high fat diet, LF = low fat diet, Fattiness = residuals of liver fat mass regressed onto lean mass, Fat Content = ratio of liver fat mass to total liver mass. Strain numbers are prefaced by LGXSM-, except strains Large (LG/J) and Small (SM/J).
